# Metal-Free 2D/2D van der Waals Heterojunction Based on Covalent Organic Frameworks for Highly Efficient Solar Energy Catalysis

**DOI:** 10.1007/s40820-023-01100-x

**Published:** 2023-05-22

**Authors:** Ge Yan, Xiaodong Sun, Yu Zhang, Hui Li, Hongwei Huang, Baohua Jia, Dawei Su, Tianyi Ma

**Affiliations:** 1 Institute of Clean Energy Chemistry, Key Laboratory for Green Synthesis and Preparative Chemistry of Adv. Mater., College of Chemistry, Liaoning University, Shenyang, 110036 People’s Republic of China; 2 School of Science, RMIT University, Melbourne, VIC 3000 Australia; 3 Faculty of Science, School of Mathematical and Physical Sciences, University of Technology Sydney, Sydney, NSW 2007 Australia; 4 Beijing Key Laboratory of Materials Utilization of Nonmetallic Minerals and Solid Wastes, National Laboratory of Mineral Materials, School of Materials Science and Technology, China University of Geosciences, Beijing, 100083 People’s Republic of China

**Keywords:** Covalent organic frameworks, 2D/2D van der Waals heterojunction, Metal-free photocatalyst

## Abstract

Covalent organic frameworks (COFs) have emerged as a kind of rising star materials in photocatalysis. However, their photocatalytic activities are restricted by the high photogenerated electron–hole pairs recombination rate. Herein, a novel metal-free 2D/2D van der Waals heterojunction, composed of a two-dimensional (2D) COF with ketoenamine linkage (TpPa-1-COF) and 2D defective hexagonal boron nitride (*h*-BN), is successfully constructed through in situ solvothermal method. Benefitting from the presence of VDW heterojunction, larger contact area and intimate electronic coupling can be formed between the interface of TpPa-1-COF and defective *h*-BN, which make contributions to promoting charge carriers separation. The introduced defects can also endow the *h*-BN with porous structure, thus providing more reactive sites. Moreover, the TpPa-1-COF will undergo a structural transformation after being integrated with defective *h*-BN, which can enlarge the gap between the conduction band position of the *h*-BN and TpPa-1-COF, and suppress electron backflow, corroborated by experimental and density functional theory calculations results. Accordingly, the resulting porous *h*-BN/TpPa-1-COF metal-free VDW heterojunction displays outstanding solar energy catalytic activity for water splitting without co-catalysts, and the H_2_ evolution rate can reach up to 3.15 mmol g^−1^ h^−1^, which is about 67 times greater than that of pristine TpPa-1-COF, also surpassing that of state-of-the-art metal-free-based photocatalysts reported to date. In particular, it is the first work for constructing COFs-based heterojunctions with the help of *h*-BN, which may provide new avenue for designing highly efficient metal-free-based photocatalysts for H_2_ evolution.

**Supplementary Information:**

The online version contains supplementary material available at 10.1007/s40820-023-01100-x.

## Highlights

- It is the first attempt to combine covalent organic frameworks with hexagonal boron nitride (*h*-BN) to construct efficient metal-free photocatalyst.
- The composite displays superior photocatalytic hydrogen production performance in metal-free systems.
- The integrated porous *h*-BN can suppress electron backflow to optimize the composite photocatalytic activity.

**Supplementary Information:**

The online version contains supplementary material available at 10.1007/s40820-023-01100-x.

## Graphical abstract

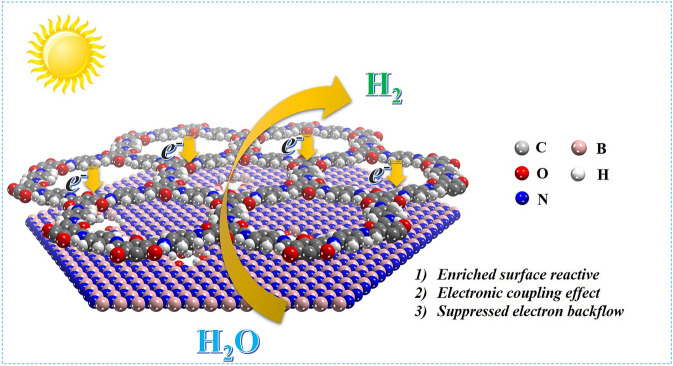

## Introduction

Considering the severe and intractable energy and environmental crises, exploring renewable energy alternatives to fossil fuels has become a hot yet challenging research topic [1–5]. Solar energy catalytic water splitting to produce H_2_ undoubtedly provides a promising avenue for solving above problems, but the key is seeking for ideal photocatalysts with highly active and permanent stability [6–26]. Covalent organic frameworks (COFs), a kind of highly crystalline porous materials constructed through condensation of organic building unit, have shown great potential in the field of photocatalytic water splitting, owing to their high specific surface area, extraordinary chemical stability, designable knots and linkers with tunable photo- and electro-active [27–37]. However, the separation efficiency and rapid charges carriers recombination are still the main drawbacks for optimizing their solar energy catalytic performance.

According to this situation, series of modification strategies, including metal doping, ligand modification, and defect engineering, have been developed [38–41]. In particular, the heterostructure constructions, which possess the advantages of synergistic effect and efficient electron–hole pairs separation, were proved to be a robust strategy to conquer above limitations for COFs. One of the most fascinating examples was reported by Lan’s group, through combining a typical Schiff-based COFs, TpPa-1-COF, and an amino-functionalized Zr-MOFs, UiO-66-NH_2_, covalently integrated MOF/COF hybrid were successfully constructed. Benefitting from the suitable band structure and covalent bonds connection between two materials, the electron–hole pairs separation/migration efficiency can be effectively promoted thus displaying a commendable sun-light-driven water splitting performance [42].Apart from above-mentioned example, other COFs-based heterojunctions with photocatalytic water splitting activity, such as COFs/CdS, COFs/WO_3_, COFs/TiO_2_ and COFs/α–Fe_2_O_3_, have also been reported [43–46]. However, most heterostructures are a combination of two-dimensional (2D) COFs and 3D semiconductor materials, which is accompanied by the issues of lattice mismatch and atomic interdiffusion, thus unfavorable for the charge transfer and separation [47–50]. Moreover, most of COFs-based heterostructures contain metals, and the non-metal COFs-based heterostructures have rarely been reported.

Recently, the emerging 2D/2D van der Waals (VDW) heterojunction, which are assembled through VDW interactions, attracts tremendous in photocatalysts designing [51, 52]. Compared with traditional heterojunctions, the superiorities of the VDW heterojunctions can be generalized as follows: (1) the face-to-face contact is beneficial for forming the strong interactions and increasing the interface area; (2) the intimate interlayer electronic interactions are favorable for optimizing electronic structure; (3) the VDW force can facilitate the high-speed charge transfer channels formation, thus accelerating the electron–hole pairs migration/separation; (4) it is undesired for considering the issue of lattice matching between the layers of composite materials. Herein, selecting the suitable 2D materials to integrate with 2D COFs to form the VDW heterojunctions can effectively enhance their charge transfer efficiency and promote the photocatalytic water splitting performance.

Hexagonal boron nitride (*h*-BN), which can also be called white graphene with honeycomb 2D structure, is composed of lightweight elements with unique physical and chemical properties. Due to the wide band gap (~ 5.0–5.6 eV), *h*-BN was not considered as ideal photocatalysts previously. In this regard, some modification methods, such as element doping, thickness regulation and defect engineering, have been recently developed to broaden the research fields of *h*-BN in photocatalysis [53]. Among them, the defect engineering strategy displays many advantages, through inducing defects; (1) new valence and conduction band can be introduced, thus narrowing the bandgap, and broadening the light absorption region of *h*-BN; (2) the porous structure can be endowed with *h*-BN, thus enriching the reactive sites, and boosting substrates transfer; (3) abundant hydroxyl (–OH) and secondary amino (–NH) groups can be generated on their surface, which can be regarded as trapping sites to connect other materials to form the heterojunctions, thus facilitating the charge transfer. Therefore, combining 2D COFs with defect engineered *h*-BN may provide opportunities for preparing novel non-metal VDW heterojunction photocatalysts.

Inspired by above, we for the first time combine a 2D COF containing ketoenamine linkage (TpPa-1-COF) and porous *h*-BN to successfully prepare a novel metal-free 2D/2D VDW heterojunction through a facile calcination–solvothermal strategy. With an optimal content proportion between TpPa-1-COF and porous *h*-BN, the VDW heterojunction can effectively promote water splitting without the addition of co-catalysts, and the H_2_ evolution rate is as high as 3.15 mmol g^−1^ h^−1^, which is one of the best performed metal-free photocatalysts. The commendable photocatalytic activity can be attributed to the porous structure of two materials and the strong electronic coupling at their interface. More interestingly, the incorporation of porous *h*-BN can also initiate the structural transformation of TpPa-1-COF, thus enhancing its conduction band position and suppressing electron backflow, which is also beneficial for promoting water splitting. This example of metal-free VDW heterojunction is expected to afford new inspirations for the further highly efficient photocatalysts design.

## Experimental Section

### Materials

All chemicals were purchased commercially and used without further purification.

### Synthesis of Porous *h*-BN, TpPa-1-COF and Porous *h*-BN/TpPa-1-COF

#### Synthesis of Porous *h*-BN

The method of preparing porous *h*-BN nanosheets followed the previous literature with slight changes [54]. First, boric acid (4 g, 0.06472 mol) was added to 80 mL of deionized water along with urea (16 g, 0.2667 mol) and heated at 80 °C for 4 h. Then, the transparent solution acquired was transferred to a pear-shaped flask and the solvent water was removed. Finally, the substance was placed in a porcelain boat and calcined at 900 °C under N_2_ atmosphere for five hours, with a heating rate of 5 °C per minute. The white object obtained was washed three times by centrifugation.

#### Synthesis of TpPa-1-COF

TpPa-1-COF was prepared according to reported works with minor modifications [55]. First, 1, 3, 5-triformylphloroglucinol (Tp) (31 mg, 0.15 mmol) and paraphenylenediamine (24 mg, Pa-1) ligands were dispersed in 3 mL *N*, *N*–dimethylformamide (DMF) by ultrasound. Then, 0.5 mL acetic acid (3 M) was added into the tube. After the solution is ultrasonic evenly, transfer the tube into liquid nitrogen (77 K) for rapid freezing, and vacuum it three times. Then, the tube was sealed and transferred into oven to heat at 120 °C for 3 days. The product was collected by centrifugation, then washed with anhydrous THF and anhydrous acetone for several times, and finally dried in vacuum at 60 °C for 24 h.

#### Synthesis of Porous *h*-BN/TpPa-1-COF

The porous *h*-BN/TpPa-1-COF heterojunction was constructed through in situ solvothermal method. First, the obtained porous *h*-BN was scattered into 3 mL of DMF. Then, adding the Tp (21 mg, 0.1 mmol), Pa-1 (17 mg, 0.15 mmol) and aqueous acetic acid (0.5 mL, 3 M) into the above porous *h*-BN solution. After that, stirring the tube and outgassed by freeze–pump–thaw for three times under liquid N_2_ (77K) freezing. Then, the above mixture was transferred into oven to heat at 120 °C for 3 days. The product was collected by centrifugation, washed with anhydrous THF and anhydrous acetone for several times, and finally dried in vacuum at 60 °C for 24 h.

### Characterizations

Scanning electron microscopy (SEM) was performed on a Hitachi SU-8010 equipped with an EDS analyzer. Transmission electron microscopy (TEM) and high-resolution TEM (HRTEM) were performed on a JEM-2100 operating at 200 kV, respectively. Fourier transform infrared (FT-IR) spectra were carried out on a Nicolet Nexus 670 FT-IR spectrophotometer by mixing KBr and the sample. Powder X-ray diffraction (PXRD, Bruker D8 Advance) was used to analyze the structure of the photocatalysts. Thermogravimetric analysis (TGA) was conducted on a METTLER TOLEDO TGA/SDTA851 in nitrogen environment with a heating rate of 5 °C min^−1^. The X-ray photoelectron spectroscopy (XPS) was conducted on England Kratos, Ultra DLD spectrometer equipped with a monochromatic Al Kα X-ray source (1486.6 eV). The UV–vis diffuse reflectance spectra (DRS) were obtained on a Shimadzu UV-2550. The surface photovoltage (SPV) test was performed at room temperature, and the PV signal of the sample was recorded under the excitation of a laser pulse (50 mJ cm^−2^).

### Photocatalytic Testing

The online system (Labsolar-6A, Beijing Perfectlight Technology Co., Ltd.) equipped with a GC7900 gas chromatography (Shanghai Tianmei Scientific Instrument Co. high-purity N_2_ as the carrier gas) was used to investigate the photocatalytic H_2_ evolution performance of the as-synthesized photocatalyst. The light source is a 300-W xenon lamp with a 420 nm filter. The actual light intensity irradiated on the reaction surface is 407.5 mW. The distance between the light and the reactor is 2 cm, and the irradiated area of the reactor is 11.0 cm^2^. The specific reaction device and reactor shape are shown in Fig. S1. The experiment was carried out in a quartz reactor. 10 mg photocatalyst was dispersed in 100 mL ultrapure water by ultrasound, and 100 mg l-ascorbic acid was added for magnetic agitation. The reaction system is condensed at 5 °C, and the reactor is vacuumed to a negative pressure before illumination. The apparent quantum efficiency (AQY) was tested by a Xe lamp equipped with bandpass filters. The usage of catalyst is 5, 10 and 20 mg. The wavelengths of the filters were 400, 420, 500, 520, and 550 nm. The specific value of AQY can be calculated by Eq. 1:

1 $${\text{AQY}} = \frac{{{\text{Ne}}}}{{{\text{Np}}}} \times 100\%$$

where Ne and Np represent the total number of electrons transferred by the reaction and the number of incident photons, respectively.

### Photoelectrochemical Testing

The CH Instruments Inc electrochemical workstation (CHI 660E) was used for electrochemical testing. The electrolyte employed in electrochemical tests was Na_2_SO_4_ (2 M), and the reference and counter electrodes (RE and CE) were Ag/AgCl and Pt wire, respectively. The samples (2 mg) with 10 µL Nafion and 1 mL ethanol were mixed to acquire liquor. Covering the fluorine-tin oxide (FTO) glass (1.5 cm × 1.5 cm) with the mixture, the FIO served as the working electrode (WE). Mott–Schottky (M-S) plots and linear sweep voltammetry (LSV) were recorded on a standard three-electrode system. Electrochemical impedance spectroscopy (EIS) measurements and photocurrent-time (I-T) profiles were recorded on a standard two-electrode system. The current transfer efficiency is calculated according to Eq. 2:

2 $$\eta_{{{\text{trans}}}} = \frac{{J_{{{\text{H}}_{2} {\text{O}}}} }}{{J_{{{\text{H}}_{2} {\text{O}}_{2} }} }}$$

where H_2_O_2_ was added to the electrolyte solution and the concentration reaches 0.5 mol L^−1^. The average lifetime of the photogenerated carriers calculated by the open-circuit voltage decay method is derived from Eq. 3:

3 $$\tau_{n} = \frac{{K_{{\text{B}}} T}}{e} \left( {\frac{{{\text{dOCVD}}}}{{{\text{d}}t}}} \right)^{ - 1}$$

where *K*_B_ as the Boltzmann constant, the value is 1.38 × 10^−23^ J K^−1^, *T* is temperature (298 K), *e* is the electric charge (1.602 × 10^−19^ C), and dOCVD/d*t* is the derivative of the OCP transient decay.

### DFT Calculations

DFT calculations were performed using the Vienna Ab initio Simulation Package [56–59] on the basis of the GGA with the Perdew–Burke–Ernzerhof [60] function as the exchange–correlation energy function. For the bulk BN crystals 4 × 4 × 1 unit cells were used for the crystal structure and electronic structure calculations. For the monolayer BN and defective monolayer BN crystals 9 × 9 × 1 unit cells were used for the crystal structure and electronic structure calculations. For the COF and defective COF, 1 × 1 × 1 unit cells were used for the crystal structure and electronic structure calculations. At least 20 Å vacuum level was used for the layer structures calculation and the D3 correction was applied for all the layer structures calculation for the consideration of the Van der Waals effect. We used the projector augmented wave potentials [61] with the cutoff energy of 450 eV. The conjugate gradient scheme is used to optimize the atom coordinates until the force is less than 0.01 eV Å^−1^. The number of k-points was carefully optimized to achieve energy convergence, giving 6 × 6 × 1 Monkhorst–Pack BZ calculations for the bulk BN calculation. Surface calculations were conducted via a slab model with periodic boundary conditions. The (001) crystal planes of bulk BN and surface with defects BN were cleaved from the relaxed bulk crystals and built with a vacuum of about 20 Å and a four-layer slab of which the one bottom layer was kept fixed and 6 × 6 × 1 k-point mesh during relaxation [62–64].

Calculation on the Conductive Bond (CB), Valence Bond (VB), and Fermi level positions.

The Fermi level versus vacuum level is firstly obtained via VASP calculation, and the Fermi level versus SHE is then calculated using the following Eq. 4:

4 $${\text{EF}}\left( {{\text{vs}}.{\text{ SHE}}} \right) \, = \, - 4.44 \, V - {\text{EF}}\left( {{\text{vs}}.{\text{ vacuum level}}} \right)$$

The CB and VB were calculated by:

5 $${\text{CB }} = {\text{ EF}}\left( {{\text{vs}}.{\text{ SHE}}} \right) + {\text{Eg}}/2$$

6 $${\text{VB }} = {\text{ CB}} - E_{{\text{g}}}$$

where *E*_g_ is the bond gap.

## Results and Discussion

### Preparation and Characterization of Catalysts

**Scheme 1:**
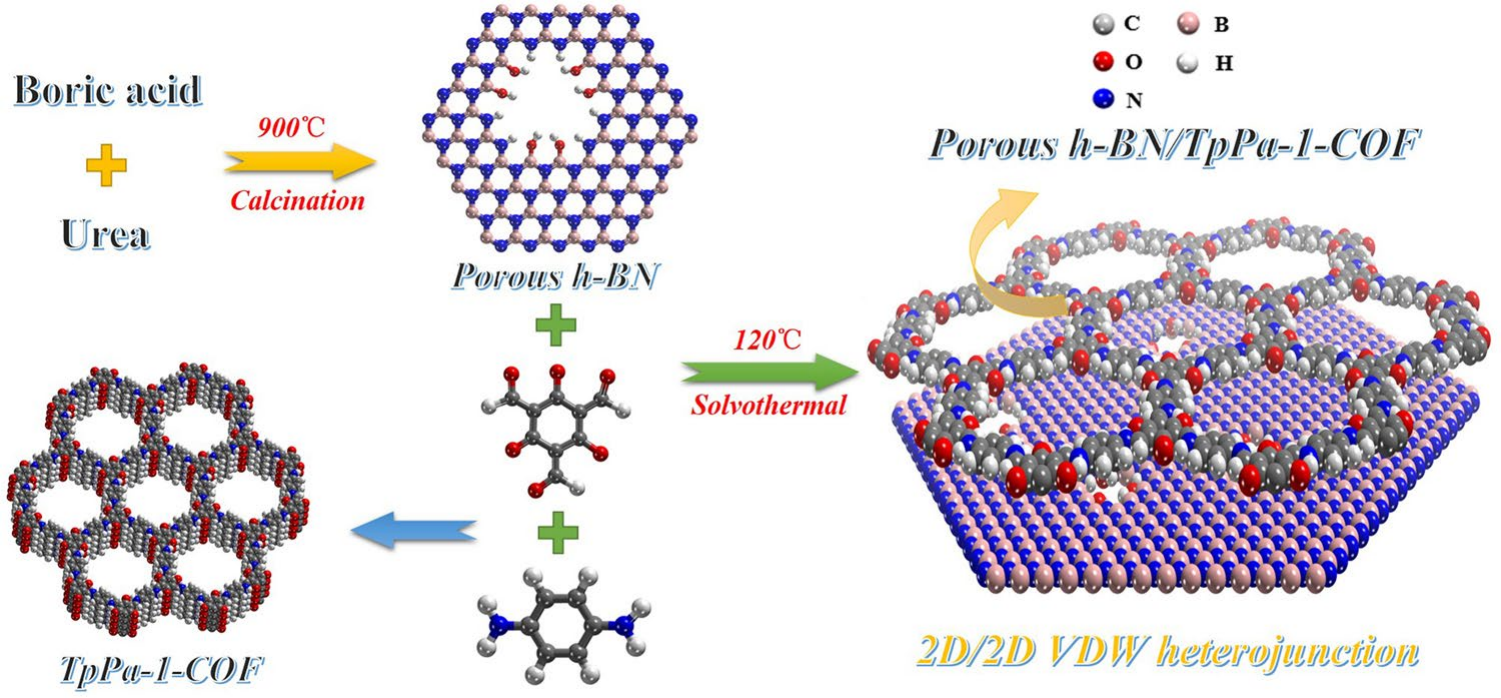
Schematic diagram of the synthesis route for porous *h*-BN/TpPa-1-COF composites

In order to obtain the COFs-based 2D/2D VDW heterojunction, a facile calcination–solvothermal strategy was employed in our work. The synthetic procedure can be illustrated in Scheme 1. By using boric acid and urea as the raw materials, the *h*-BN was successfully constructed through a high temperature calcination method, in which the high temperature can induce defects and endow *h*-BN with porous structure. After that, different amount of obtained porous *h*-BN samples were added into the synthetic system of the 2D β-Ketoenamine-linked COFs (TpPa-1-COF) to directly acquire the porous *h*-BN/TpPa-1-COF composites with different mix proportion (5%, 7.5%, 10%, 15% and 20% *h*-BN/TpPa-1-COF) through a simple solvothermal step.

**Fig. 1:**
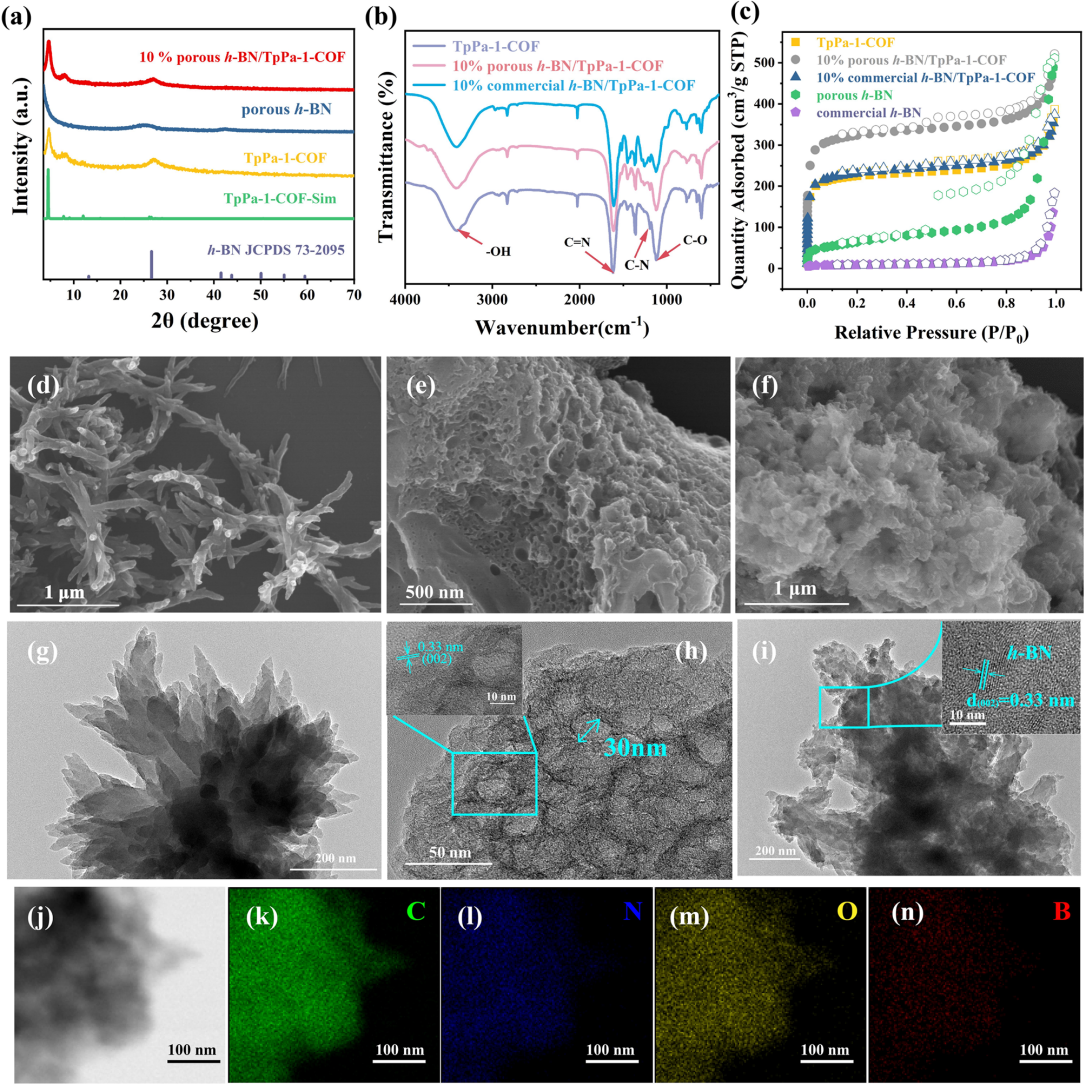
**a** PXRD patterns of TpPa-1-COF, porous *h*-BN, 10% porous *h*-BN/TpPa-1-COF, simulated TpPa-1-COF and standard *h*-BN. **b** FT-IR spectra of TpPa-1-COF, 10% commercial *h*-BN/TpPa-1-COF and 10% porous *h*-BN/TpPa-1-COF. **c** N_2_ adsorption–desorption isotherms of TpPa-1-COF, 10% commercial *h*-BN/TpPa-1-COF, 10% porous *h*-BN/TpPa-1-COF, porous *h*-BN and commercial *h*-BN. **d–f** SEM images of TpPa-1-COF, porous *h*-BN, 10% porous *h*-BN/TpPa-1-COF, 10% commercial *h*-BN/TpPa-1-COF. **g–i** TEM images for TpPa-1-COF, 10% porous *h*-BN/TpPa-1-COF and porous *h*-BN, and the inset images of (**h, i**) show the HRTEM of 10% porous *h*-BN/TpPa-1-COF and porous *h*-BN. **j–n** EDS mapping images for 10% porous *h*-BN/TpPa-1-COF

To unveil the phase purity and successful preparation of porous *h*-BN/TpPa-1-COF heterojunction, the powder X-ray diffraction (PXRD) tests were implemented. As depicted in Fig. 1a, the experimental PXRD pattern of the TpPa-1-COF is in good agreement with the simulated patterns and displays two obvious diffraction peaks at 4.6° and 26.6°, corresponding to the (100) and (001) planes of TpPa-1-COF, respectively [65]. In addition, the porous *h*-BN exhibits a broad diffraction peak around 26°, which belongs to its (002) crystal plane (JCPDS No. 73-2095). The above results proved that two pure substances were successfully obtained. In the PXRD pattern of porous *h*-BN/TpPa-1-COF composite, the diffraction peaks of TpPa-1-COF can be clearly observed. Nevertheless, due to a relative low diffraction intensity and approximate diffraction peak positions between TpPa-1-COF and *h*-BN, the sign of *h*-BN diffraction peak is difficult to distinguish. However, the presence of *h*-BN can be indicated through further experimental results. Then, the FT-IR spectra were recorded for commercial *h*-BN, porous *h*-BN, TpPa-1-COF and their composites. In comparison with commercial *h*-BN, porous *h*-BN displays a relative broader peak around 1400 cm^−1^, assigned to the stretching vibration of in-plane B-N bond, and the broadened peaks can be attributed to the enhanced B-N vibration spread. The out-of-plane B–N–B bond of porous *h*-BN displays a red shift at approximate 780 cm^−1^, which is caused by the dislocation and disruption of the *h*-BN lattice. Moreover, the presence of hydroxyl and imine groups on the surface of porous *h*-BN can also be demonstrated by the characteristic peaks at about 3200 and 1600 cm^−1^, respectively (Fig. S4) [66]. On the other hand, the stretching vibration peaks of C=N, C–N, and C–O bonds can be observed at 1617, 1191, 1115 cm^−1^, further proving the successful preparation of TpPa-1-COF.

To identify the permanent porosity of the samples, the nitrogen sorption isotherm at 77 K was proceeded. As illustrated in Fig. 1c, the commercial *h*-BN is a kind of non-porous material, and through a high temperature calcination treatment, the defects can be produced thus endowing *h*-BN with mesoporous pores, in which the pore size is estimated to be around 30 nm with a Brunauer–Emmett–Teller (BET) surface area of 231 m^2^ g^−1^ (Fig. S7). The TpPa-1-COF displays a fully reversible type I curve with high N_2_ uptake capacity at relatively low pressure, which indicates its microporous feature with a BET surface area of 743 m^2^ g^−1^. As revealed by the pore size distributions results, the pore size of TpPa-1-COF is approximate 1.2 nm, which is in accordance with their crystal structure. It is interesting that through combining TpPa-1-COF and porous *h*-BN, the BET surface area is increased to 1073 m^2^ g^−1^. The above result can be illustrated by the enriched microporous channel at about 0.6 nm, demonstrated by the pore size distribution of porous *h*-BN/TpPa-1-COF. The improved BET surface area of composites is beneficial for enriching the active sites, thus promoting the reaction of photocatalytic H_2_ production. The thermal stability of materials was explored through thermogravimetric (TG) analysis. As shown in Fig. S10, the decomposition temperature of TpPa-1-COF is about 425 °C and reveals a relative high thermal stability. After combining two materials, the decomposition temperature of composite is basically consistent with the pristine TpPa-1-COF, which prove that the addition of *h*-BN will not break the framework of TpPa-1-COF. Moreover, based on the weight loss, the proportion of two materials is able to be ascertained.

The morphology and microstructures of the products were observed by SEM and TEM images. As observed in Fig. 1d, g, the TpPa-1-COF displays a flower-like morphology formed by stacking nanorods. Compared with commercial *h*-BN with solid flake structure, the defective *h*-BN possesses highly ordered mesoporous channels, and the pore size is measured to be 30 nm (Fig. 1h and inset image), which is in agreement with the pore size distributions result. The porous structure provides more contact area and improves the electronic coupling effect with TpPa-1-COF. As shown in Fig. 1f, i, TpPa-1-COF is uniformly grown on the surface of defective *h*-BN and covers the mesoporous channels. The lattice fringe of porous *h*-BN can be distinctly seen in high-resolution transmission electron microscope (HRTEM) image of the composite, in which the value is 0.33 nm, corresponding to the (002) crystal plane. Moreover, the energy dispersive spectroscopy (EDS) elemental mapping images demonstrate that B, N, C and O elements are uniformly distributed in the composites, and the unapparent B element image can be attributed to the overlap of TpPa-1-COF on porous *h*-BN. All above results demonstrate that porous *h*-BN and TpPa-1-COF are well-integrated (Fig. 1j-n).

### XPS and Structural Analysis

**Fig. 2:**
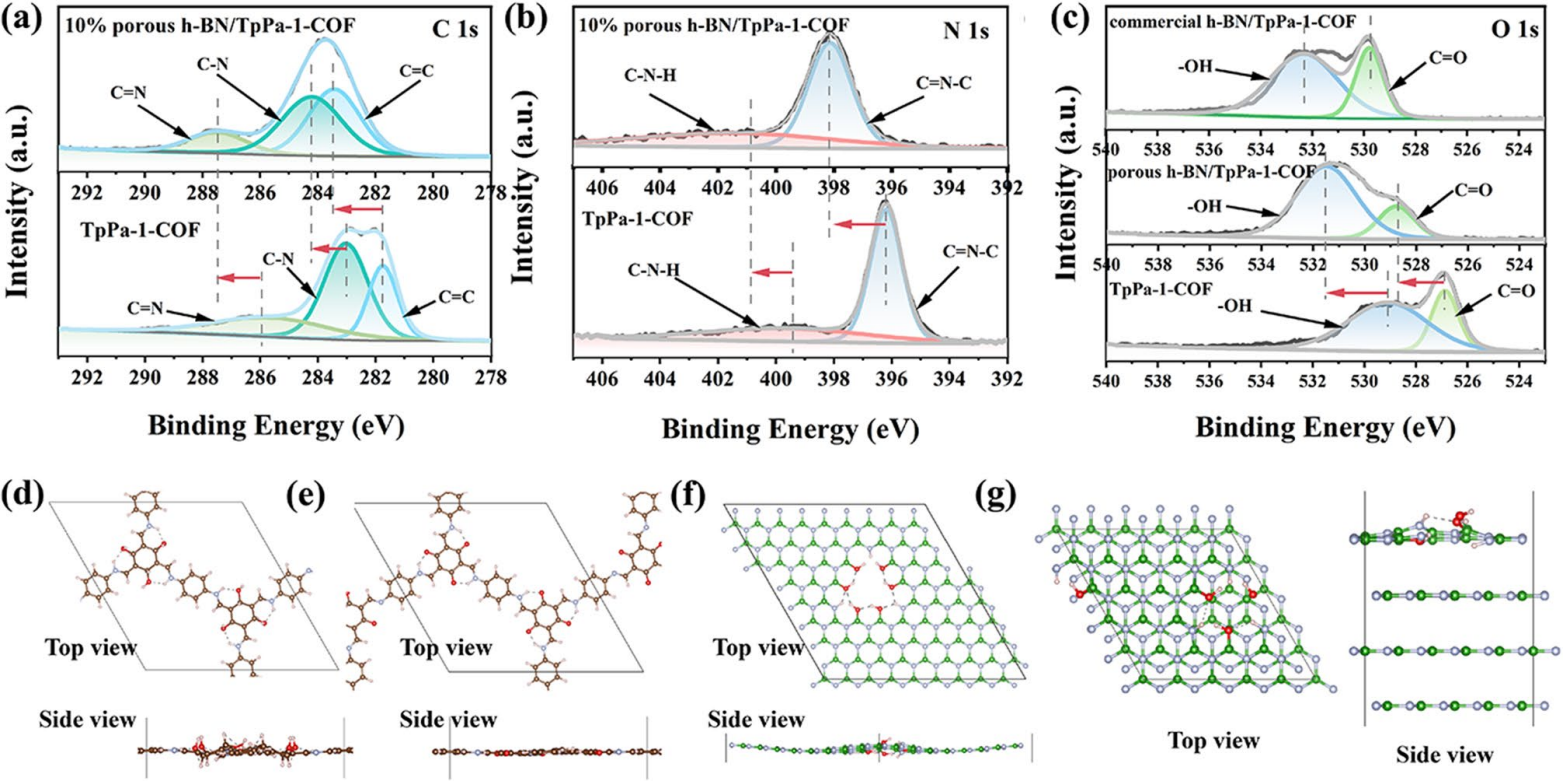
High-resolution spectra of 10% porous* h*-BN/TpPa-1-COF and TpPa-1-COF: **a** C 1*s* and **b** N 1*s*, **c** high-resolution O 1*s* XPS spectra for 10% commercial* h*-BN/TpPa-1-COF, 10% porous* h*-BN/TpPa-1-COF and TpPa-1-COF. DFT relaxed structure of transformed **d** TpPa-1-COF, **e** TpPa-1-COF, **f** defective monolayer *h*-BN, and **g** bulk BN with defective surface

XPS tests were performed to explore the chemical states and surface elemental composition of the samples. It can be observed from Fig. S18 that 10% porous *h*-BN/TpPa-1-COF contains C, N, B and O elements without extra elements, which is consistent with EDS mapping results. Furthermore, in the high-resolution C 1*s* spectrum of TpPa-1-COF, there exist four peaks located at 281.7, 283, 285.8 and 289.1 eV, corresponding to C=C, C–N, C=N and C=O bonds, respectively (Fig. 2a). Moreover, the presence of imine bond can also be revealed by the high-resolution N 1*s* spectrum, in which two N 1*s* core-level peaks are observed at 396.2 and 399.7 eV, matched up to the C=N–C and C–N–H bonds, respectively **(**Fig. 2b**)** [67]. The above results demonstrate the successful construction of TpPa-1-COF. Compared with commercial *h*-BN, porous *h*-BN displays an obvious B–O peak at 189.1 eV in high-resolution B 1*s* spectrum, and a N–H peak at 396.7 eV, verified the generation of hydroxyl and imine groups on porous *h*-BN (Figs. S19 and S20) [66]. The strong electronic coupling between two materials can also be proved by the XPS results. In comparison with pristine TpPa-1-COF, the high-resolution C 1*s*, N 1*s* and O 1*s* spectrum of porous *h*-BN/TpPa-1-COF all display obvious positive shift of binding energy induced by the presence of VDW interactions, also suggesting that the strong electronic coupling can promote electrons transfer from TpPa-1-COF to porous *h*-BN. Compared with TpPa-1-COF and commercial *h*-BN/TpPa-1-COF samples, the as-prepared porous *h*-BN/TpPa-1-COF sample’s high-resolved O 1s XPS peaks show the obvious intensity difference at around 592 and 531 eV, corresponding to C=O and O–H, respectively. It is indicated that when involving the porous *h*-BN for the TpPa-1-COF synthesis, TpPa-1-COF will form more hydroxy groups (Fig. 2c**)**. Therefore, we compared the TpPa-1-COF with the aldehyde and hydroxy group via the density functional theory. Figure 2d,e is the corresponding relaxed unit cell of TpPa-1-COF with the hydroxy and aldehyde group, respectively. It can be seen that due to the double bond break of the aldehyde group and the single bond between the carbon and hydroxyl group, the conjugated carbon ring was destroyed, while the cyclohexane was formed. Furthermore, the O 1*s* XPS peaks of porous *h*-BN and commercial *h*-BN indicate they have a comparable magnitude of the hydroxy group. We further calculated the monolayer porous *h*-BN and bulk commercial *h*-BN with the hydroxyl group on the B and proton on the N (Fig. 2f, g). The DFT relaxed structures show the natural curved architecture of the monolayer porous *h*-BN and the top layer of the bulk BN. These can be readily ascribed to the hydroxy and proton defects.

### Energy Band Structure and Performance Analysis

**Fig. 3:**
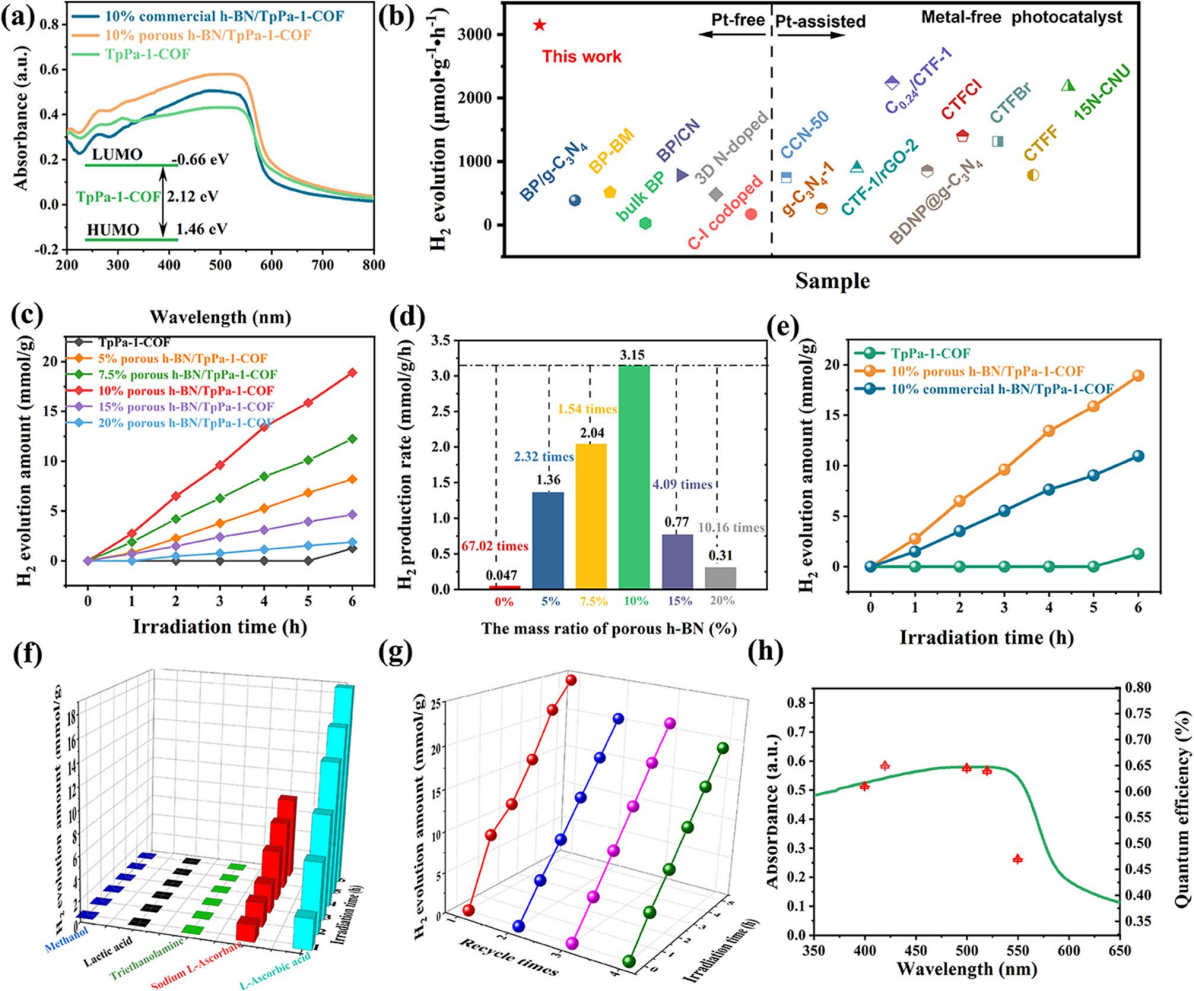
**a** DRS spectra of 10% commercial *h*-BN/TpPa-1-COF, 10% porous *h*-BN/TpPa-1-COF and TpPa-1-COF. **b** Photocatalytic hydrogen production rate of 10% porous *h*-BN/TpPa-1-COF and the reported metal-free photocatalysts. **c** Photocatalytic H_2_ evolution amount comparison with time for different ratios of porous *h*-BN/TpPa-1-COF. **d** Hydrogen production rate of a series of porous *h*-BN/TpPa-1-COF in six hours. **e** Photocatalytic hydrogen production performance of 10% porous *h*-BN/TpPa-1-COF, 10% commercial *h*-BN/TpPa-1-COF and TpPa-1-COF. **f** Photocatalytic hydrogen evolution efficiency of 10% porous *h*-BN/TpPa-1-COF under different sacrificial systems. **g** Recycling performance of 10% porous *h*-BN/TpPa-1-COF. **h** Wavelength-dependent apparent quantum efficiency (AQE) of 10% porous *h*-BN/TpPa-1-COF

The light absorption properties of the products are examined by ultraviolet–visible diffuse reflectance spectra (UV–vis DRS). As observed in Fig. 3a, the maximum absorption wavelength of TpPa-1-COF is about 610 nm, suggesting its great potential for visible light catalysis. After decorating porous *h*-BN, the light absorption edge of the composite has hardly changed, demonstrating the unchanged bandgap of TpPa-1-COF. Then, based on the Tauc diagram of Kubelka–Munk equation, the band gap energies of the TpPa-1-COF, porous *h*-BN and commercial *h*-BN can be determined to be 2.12, 4.34 and 5.7 eV, respectively [68]. The introduced defects can reduce the bandgap of *h*-BN, thus endowing it with semiconductor property. Mott–Schottky tests were also executed to determine the Fermi energy level of the samples, and further to determine their band structure [69]. The experimental results showed that the Fermi energy level of commercial *h*-BN, porous *h*-BN and TpPa-1-COF is − 0.36, − 0.38 and − 0.59 V versus Ag/AgCl, respectively (Figs. S28–S30). According to the *E*(NHE) = *E*(Ag/AgCl) + 0.197 V equation, it can be obtained that the normal hydrogen electrode (NHE) of commercial* h*-BN, porous *h*-BN and TpPa-1-COF is − 0.163, − 0.183 and − 0.393 V, respectively. Based on above results, the conduction band (CB) position of TpPa-1-COF, commercial *h*-BN and porous *h-*BN is estimated to be − 0.69, − 0.46 and − 0.48 V, respectively. Correspondingly, the valence band (VB) positions of TpPa-1-COF, commercial *h*-BN and porous *h*-BN are 1.43, 5.24 and 3.86 V, respectively. Benefitting from the suitable energy band position, the composite may be a good candidate for photocatalytic water splitting.

To corroborate our assumption, the photocatalytic hydrogen production experiments were implemented under visible light irradiation with l-ascorbic acid as sacrificial agent. In particular, the whole photocatalytic hydrogen production system was carried out in a metal-free condition. Due to the long-lasting issue of severe photoinduced electron–hole pairs recombination, the photocatalytic activity of the pristine TpPa-1-COF is poor without the addition of noble metal Pt. It is interesting that when TpPa-1-COF is combined with porous *h*-BN, their photocatalytic performance is significantly enhanced., With an optimal weight ratio, 10% porous *h*-BN/TpPa-1-COF displays the highest H_2_-evolution rate of 3.15 mmol g^−1^ h^−1^, which is 67.02 times than that of pristine TpPa-1-COF (Fig. 3c, d). It is commendable that such value can almost rank first in reported metal-free photocatalysts without the addition of noble metal Pt and also surpass most metal-free photocatalysts with the addition of noble metal Pt (Fig. 3b). To verify the important role of mesoporous channels in *h*-BN, the result of 10% commercial *h*-BN/TpPa-1-COF is also represented in Fig. 3e, where the H_2_ production rate is estimated to be 1.82 mmol g^−1^ h^−1^, that is significantly lower than 10% porous *h*-BN/TpPa-1-COF. The enhanced photocatalytic can be due to the enhanced porosity and the regulated band structure. In addition, the photocatalytic H_2_ evolution tests with different experimental conditions, such as the photocatalyst mass and the sacrificial agent, have also been performed [70–73]. As shown in Fig. S31, when the mass of used photocatalyst is 5 mg, the hydrogen production rate of the composite can reach up to 2.59 mmol g^−1^ h^−1^. Through further increase the mass of used photocatalyst, their catalytic activity increased. However, when the mass of used photocatalyst reaches to 20 mg, their catalytic activity decreased, which may be due to the shielding effect. Therefore, the optimal amount of used catalyst is 10 mg. In addition, the photocatalytic hydrogen production tests under different sacrificial agents (SEDs) were also performed, and the specific results are displayed in Fig. 3f. When selecting methanol, lactic acid, triethanolamine as the SEDs, only trace amount of H_2_ can be detected. However, by changing the SEDs to sodium l-ascorbate and l-ascorbic acid, the hydrogen production rate was significantly enhanced, especially the l-ascorbic acid can most effectively promote the hydrogen production. Moreover, the 10% porous *h*-BN/TpPa-1-COF displays an apparent quantum efficiency (AQE) of 0.65% at the wavelength of 420 nm (Fig. 3h). In addition to the catalytic activity, stability is also an important index for photocatalysts. The hydrogen-producing activity of 10% porous *h*-BN/ TpPa-1-COF showed a steady increase within 20 h. Within twenty hours, the activity increased slightly faster in the first six hours, and the rate of photocatalytic hydrogen production increased steadily in the later hours, but the high activity was still maintained, indicating the superior stability of 10% porous *h*-BN/TpPa-1-COF** (**Fig. 3g**)**. Meanwhile, combining the XRD and SEM characterizations of the cycling samples, their reusability can also be revealed (Figs. S41 and S42).

### Mechanistic Investigation

**Fig. 4:**
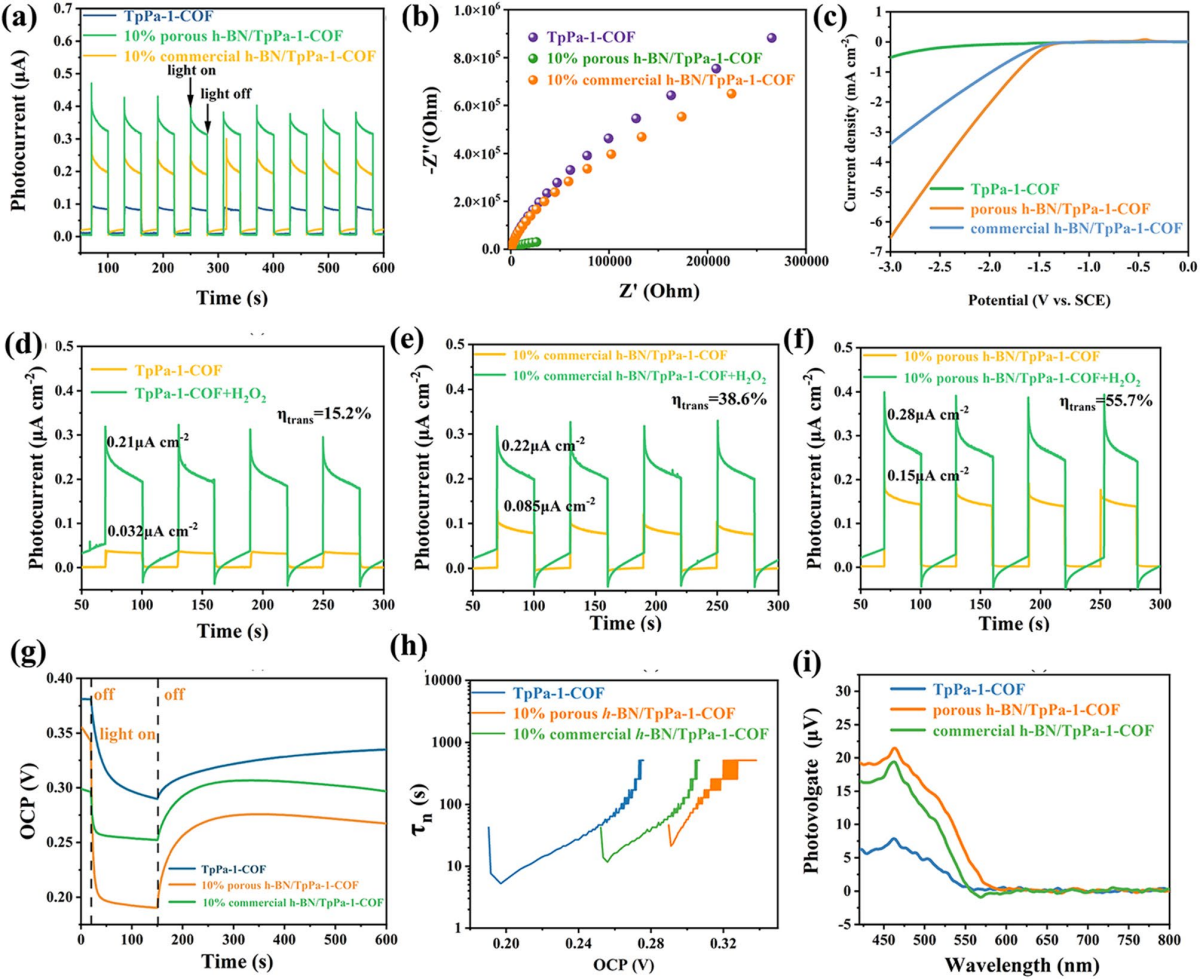
**a** Transient photocurrent response, **b** EIS Nyquist plots and **c** LSV curves of 10% porous *h*-BN/TpPa-1-COF, 10% commercial *h*-BN/TpPa-1-COF and TpPa-1-COF. **d–f** Photocurrent response of without and with adding H_2_O_2_ into electrolyte of TpPa-1-COF, 10% commercial h-BN/TpPa-1-COF and 10% porous h-BN/TpPa-1-COF. **g** OCVD curves of TpPa-1-COF, 10% commercial *h*-BN/TpPa-1-COF and 10% porous *h*-BN/TpPa-1-COF. **h** Average lifetime of the photogenerated carriers (*τ*_*n*_) for TpPa-1-COF, 10% commercial *h*-BN/TpPa-1-COF and 10% porous *h*-BN/TpPa-1-COF. **i** SPV spectra of TpPa-1-COF, 10% commercial *h*-BN/TpPa-1-COF and 10% porous *h*-BN/TpPa-1-COF

To better understand the charge carriers migration and separation properties, photoelectrochemical properties of the prepared samples were analyzed. As depicted in Fig. 4a, the photocurrent density of 10% porous *h*-BN/TpPa-1-COF is much higher than that of pristine TpPa-1-COF and 10% commercial *h*-BN/TpPa-1-COF, implying that the integrated *h*-BN is beneficial for improving the charges carriers separation, and the introduced defects can narrow the CB gap between *h*-BN and TpPa-1-COF, thus further enhancing the separation efficiency.

**Fig. 5:**
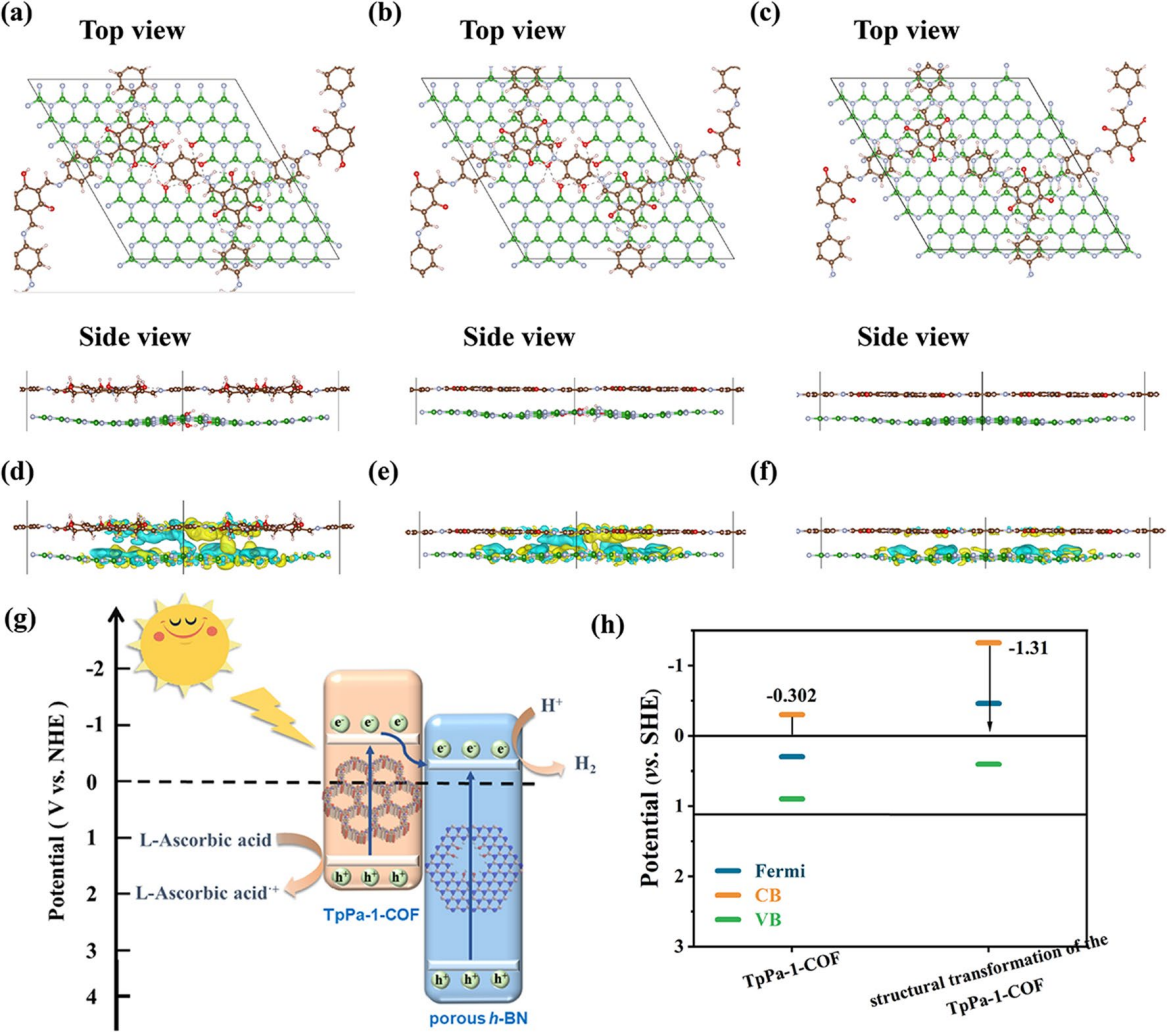
DFT relaxed structure of **a** structural transformed TpPa-1-COF and defective monolayer *h*-BN heterojunction, **b** TpPa-1-COF and defective monolayer *h*-BN heterojunction, and **c** TpPa-1-COF and monolayer BN. **d–f** The corresponding electron density isosurface = 0.0002|e|/Bohr^3^). The colored regions from turquoise to yellow represent the loss and gain of electrons, respectively. **g** Mechanism schematic of photocatalytic H_2_ evolution of porous *h*-BN/TpPa-1-COF composite materials under visible-light irradiation. **h** DFT calculated electronic bond structures. (Color figure online)

On the other hand, the resistance of electron transfer in the materials was also explored by EIS tests. The 10% porous *h*-BN/TpPa-1-COF sample exhibits the smallest semicircular diameter of Nyquist curves, revealing that the electron transfer efficiency is significantly improved (Fig. 4b). As demonstrated in linear sweep voltammetry (LSV) curves, the hydrogen production potential follows the sequence of 10% porous *h*-BN/TpPa-1-COF > 10% commercial *h*-BN/TpPa-1-COF > TpPa-1-COF, which is in accordance with the photocatalytic results (Fig. 4c). The surface charge transfer efficiency was also used to characterize the electron transfer capability of materials. The surface charge transfer efficiency can be obtained by comparing the current values of the materials with pure electrolyte and the electrolyte with the addition of H_2_O_2_ (0.5 mol L^−1^). The surface charge transfer efficiency can be obtained by comparing the current values of the materials with pure electrolyte and the electrolyte with the addition of H_2_O_2_ (0.5 mol L^−1^), where H_2_O_2_ is acting as a hole trap, making the surface charge transfer very fast and can be approximated as 100%. Therefore, according to the defining formula of photocurrent, we can finally get that the ratio of photocurrents under two different conditions, which can be taken as the surface charge transfer efficiency. As shown in Fig. 4d–f, it can be extracted that 10% porous *h*-BN/TpPa-1-COF exhibits the highest surface charge transfer efficiency, which can reach up to 55.7%. Figure 4g displays the open-circuit voltage decay curves of the samples, and its data are calculated to acquire the average lifetime of the photogenerated carriers. It can be estimated that 10% porous *h*-BN/TpPa-1-COF has the largest average lifetime, which proves that its photogenerated carriers have the lowest recombination rate (Fig. 4h). Apart from above results, the higher charge carriers dissociation efficiency can also be proved by the steady-state surface photovoltage (SPV) and transient-state surface photovoltage (TPV) results, in which the 10% porous *h*-BN/TpPa-1-COF sample all displays the highest photovoltage response signal (Figs. 4i and S45). To demonstrate the important role of porous *h*-BN in the composite, we calculated the charge density differences between the structural transformed TpPa-1-COF (with the hydroxyl group, cyclohexane) and the pristine TpPa-1-COF with the defective monolayer porous *h*-BN and monolayer *h*-BN without defects. As shown in Fig. 5a–c, the relaxed structures of the heterojunction structure of the structural transformed TpPa-1-COF and defective monolayer porous *h*-BN demonstrated the largest interlayer distance (side view of Fig. 5a). For the monolayer *h*-BN without defects, though there are no defects, it still presents curved architecture due to the interaction with the TpPa-1-COF. From their corresponding charge density difference plots (Fig. 5d, e), it can be identified that due to structural transformation of TpPa-1-COF and the defect on porous *h*-BN, there exists electronic coupling between two materials (Fig. 5d, e) by comparing with the TpPa-1-COF and *h*-BN without defects (Fig. 5f). Further comparing the TpPa-1-COF with structural transformation to TpPa-1-COF without structural transformation, the charge density difference is slightly lower, and TpPa-1-COF mainly loses the electron and transfers to defective porous monolayer *h*-BN (Fig. 5e), which facilitates the electron and hole charge separation when they form the heterojunction structure after absorption of the visible light. The photocatalysis HER working mechanism was further analyzed through electronic bond structure calculations (Fig. 5h). When considering that all oxygen in TpPa-1-COF exists in the form of carbonyl group, the CB position of TpPa-1-COF was calculated to be − 0.302 eV versus SHE. However, through combining TpPa-1-COF with porous *h*-BN, more hydroxyl groups will be formed and the TpPa-1-COF will undergo a structural transformation. In this regard, the CB position of the structural transformed TpPa-1-COF was dramatically elevated to − 1.31 eV versus SHE. Due to the elevated CB position of TpPa-1-COF, the gap between the conduction band position of the *h*-BN and TpPa-1-COF was enlarged, which is beneficial for suppressing the electron backflow.

Based on the band structure analysis and series of photoelectrochemical tests, the photocatalytic H_2_ evolution mechanism of the porous *h*-BN/TpPa-1-COF can be proposed. In virtues of the excellent visible-light absorption ability, the TpPa-1-COF is excited to generate electron–hole pairs. Thanks to the formed VDW heterojunction, the electrons on TpPa-1-COF can be facilitated by the strong electronic coupling effect to transfer to porous *h*-BN, thus achieving the photogenerated electron–hole pairs separation. Lastly, the electrons will migrate to the surface reactive sites of porous *h*-BN to reactive with H^+^ and produce H_2_. Benefitting from the introduced defect, the *h*-BN can be endowed with porous structure, thus providing more surface reactive sites. Moreover, the defect can also induce the structural transformation of the TpPa-1-COF, thus elevating its CB position and enlarging the gap between the CB position of the *h*-BN and TpPa-1-COF, which can make contribution to inhibiting the electrons backflow (Fig. 5g). Therefore, the porous *h*-BN/TpPa-1-COF sample exhibits a higher photocatalytic activity both than TpPa-1-COF and commercial *h*-BN/TpPa-1-COF samples.

## Conclusions

In conclusion, through integrating COFs and porous *h*-BN, we have successfully constructed a novel metal-free 2D/2D VDW heterojunction through a successive calcination–solvothermal strategy. In comparison with pristine COFs, the photocatalytic activity of VDW heterostructure is significantly improved with a record-breaking H_2_ evolution rate (3.15 mmol g^−1^ h^−1^) in metal-free photocatalysts with no addition of co-catalysts. Such remarkable photocatalytic performance origins from the enriched surface reactive sites, strong electronic coupling effect, and enlarged conduction band gap between COFs and porous *h*-BN, verified by series of experimental and theoretical calculation results. Moreover, this work is the first attempt to combine COFs with *h*-BN to construct lightweight metal-free photocatalysts, which may provide new insight for metal-free photocatalysts construction.

### Supplementary Information

Below is the link to the electronic supplementary material.

Supplementary file1 (PDF 4817 KB)
