# Supplementary material for: Metal-Free 2D/2D van der Waals Heterojunction Based on Covalent Organic Frameworks for Highly Efficient Solar Energy Catalysis
Source: Nanomicro Lett. 2023 May 22;15:132. doi: 10.1007/s40820-023-01100-x (PMC10200743; doi:10.1007/s40820-023-01100-x)
Supplement: Supplementary file 1 — Supplementary file1 (PDF 4817 KB) [file 40820_2023_1100_MOESM1_ESM.pdf]

Supporting Information for

Metal-Free 2D/2D VDW Heterojunction based on Covalent Organic Frameworks for Highly Efficient Solar Energy Catalysis

Ge Yan[1], Xiaodong Sun[1, *], Yu Zhang[1], Hui Li[2], Hongwei Huang[4], Baohua Jia[2], Dawei Su[3, *], Tianyi Ma[2, *]

[1]Institute of Clean Energy Chemistry, Key Laboratory for Green Synthesis and Preparative Chemistry of Adv. Mater., College of Chemistry, Liaoning University, Shenyang 110036, People's Republic of China

[2]School of Science, RMIT University, Melbourne, VIC 3000, Australia

[3]School of Mathematical and Physical Sciences, Faculty of Science, University of Technology Sydney, Sydney, NSW, 2007 Australia

[4]Beijing Key Laboratory of Materials Utilization of Nonmetallic Minerals and Solid Wastes, National Laboratory of Mineral Materials, School of Materials Science and Technology, China University of Geosciences, Beijing, 100083, People's Republic of China

*Corresponding authors. E-mail: sunxiaodong@lnu.edu.cn (Xiaodong Sun), dawei.su@uts.edu.au (Dawei Su), tianyi.ma@rmit.edu.au (Tianyi Ma)

Supplementary Figures and Tables

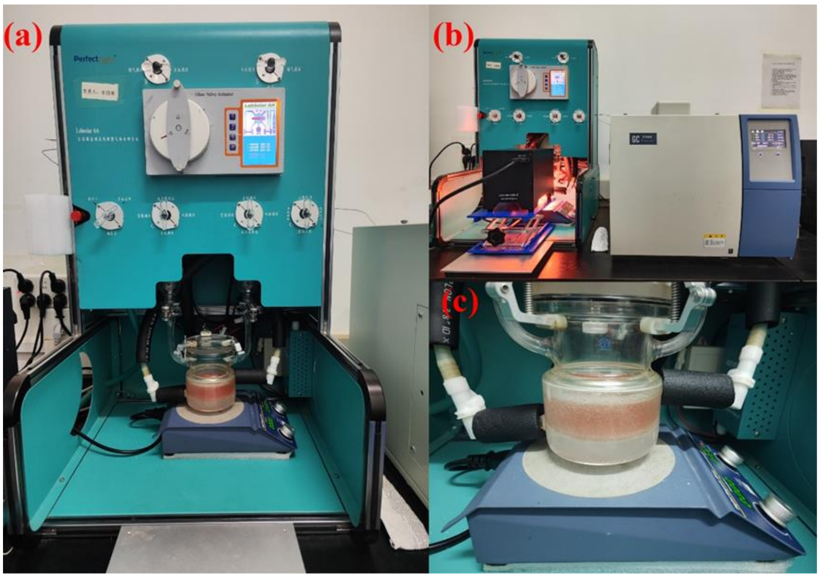

Fig. S1 (**a**) The connection of *h*-BN/TpPa-1-COF to Labsolar-6A automatic real-time trace gas analysis system of the main instrument before reaction. (**b**) The main devices required for the photocatalytic hydrogen production reaction of *h*-BN/TpPa-1-COF. (**c**) Reactor for photocatalytic reaction of *h*-BN/TpPa-1-COF

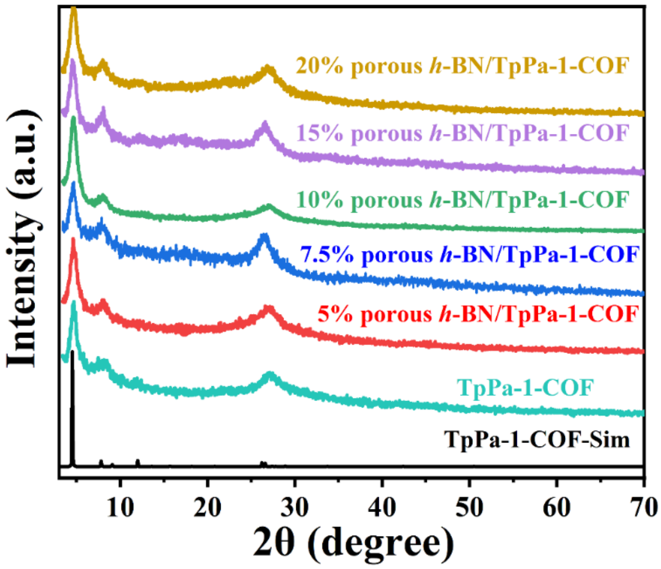

Fig. S2 Powder X-ray diffraction patterns of simulated TpPa-1-COF, TpPa-1-COF and a range of porous *h*-BN/TpPa-1-COF in different proportions. The diffraction peaks occurring at 4.6° and 26° for the simulated TpPa-1-COF, also seen in TpPa-1-COF and porous *h*-BN/TpPa-1-COF, indicated that the successful synthesis of porous *h*-BN/TpPa-1-COF and the integration with porous *h*-BN did not affect the structure of TpPa-1-COF.

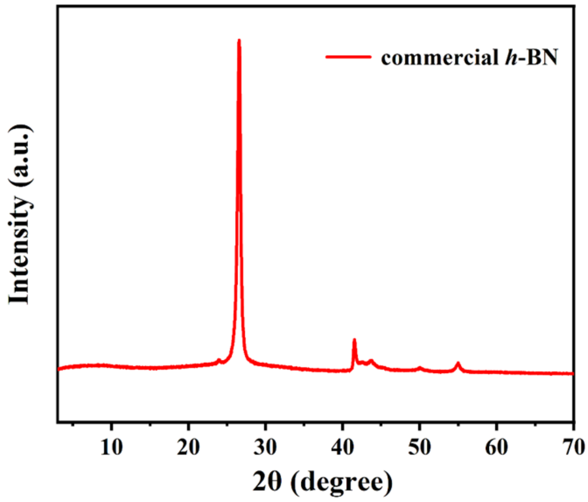

Fig. S3 Powder X-ray diffraction patterns of commercial *h*-BN, commercial *h*-BN has a distinct diffraction peak at 26°, corresponding to the (002) crystal plane. According to the comparison of PXRD results, porous *h*-BN and commercial *h*-BN have the same peak position, but porous *h*-BN is significantly less crystalline than commercial *h*-BN due to the presence of defects.

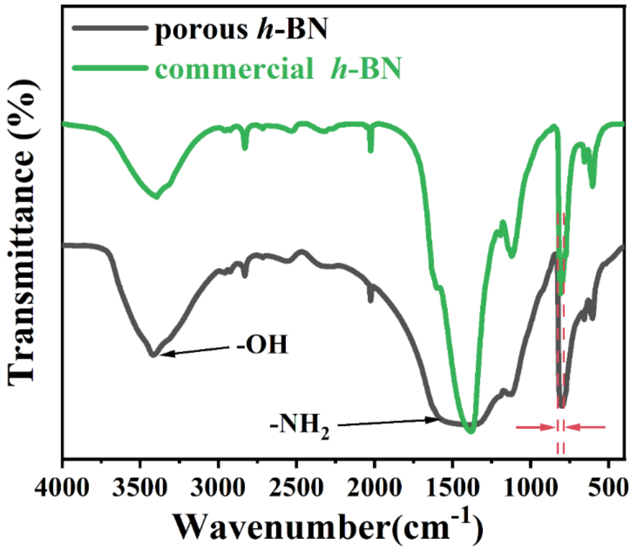

Fig. S4 FT-IR spectra of porous *h*-BN and commercial *h*-BN. Porous *h*-BN displays a relative broader peak around 1400 cm^{-1} than commercial *h*-BN, assigned to the stretching vibration of in-plane B-N bond, and the broadened peaks can be attributed to the enhanced B-N vibration spread. The out-of-plan B-N-B bond of porous *h*-BN displays a red shift at approximate 780 cm^{-1}, which is caused by the dislocation and disruption of the *h*-BN lattice. Moreover, the presence of hydroxyl and imine groups on the surface of porous *h*-BN can also be demonstrated by the characteristic peaks at about 3200 and 1600 cm^{-1}, respectively

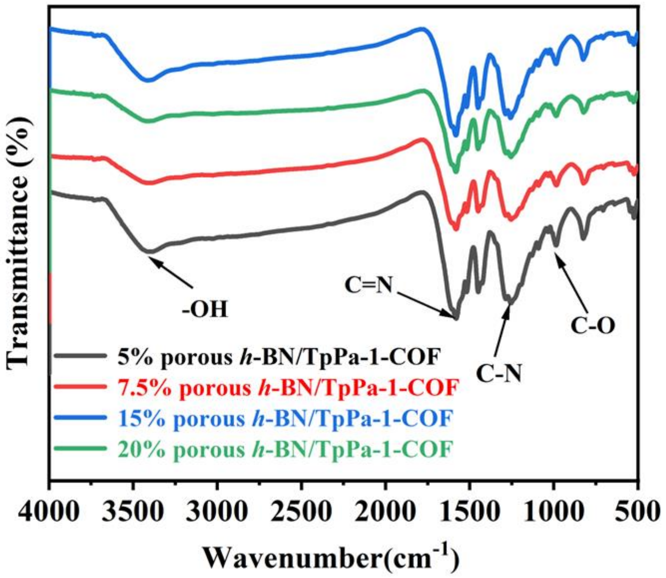

Fig. S5 FT-IR spectra of 5% porous *h*-BN/TpPa-1-COF, 7.5% porous *h*-BN/TpPa-1-COF, 15% porous *h*-BN/TpPa-1-COF and 20% porous *h*-BN/TpPa-1-COF

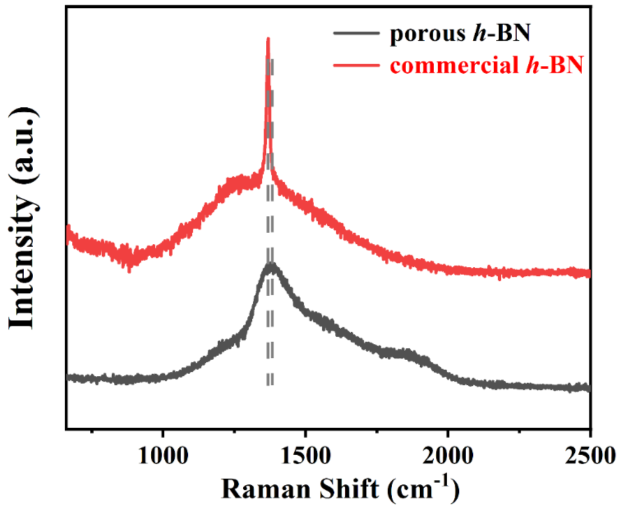

Fig. S6 Raman spectrum of porous *h*-BN and commercial *h*-BN, the high-frequency E_{2g} mode value of commercial *h*-BN is 1367 $cm^{-1}$and there is an upshift about 10 cm^{-1} for porous *h*-BN compared to the commercial *h*-BN. In addition, the full width at half maximum (FWHM) of porous *h*-BN is larger than commercial *h*-BN [S12-S15]. Combined with the previously reported literature [S16], we can know that the obtained microcrystalline size of porous *h*-BN is small with poor crystalline because of the introduced defects in *h*-BN

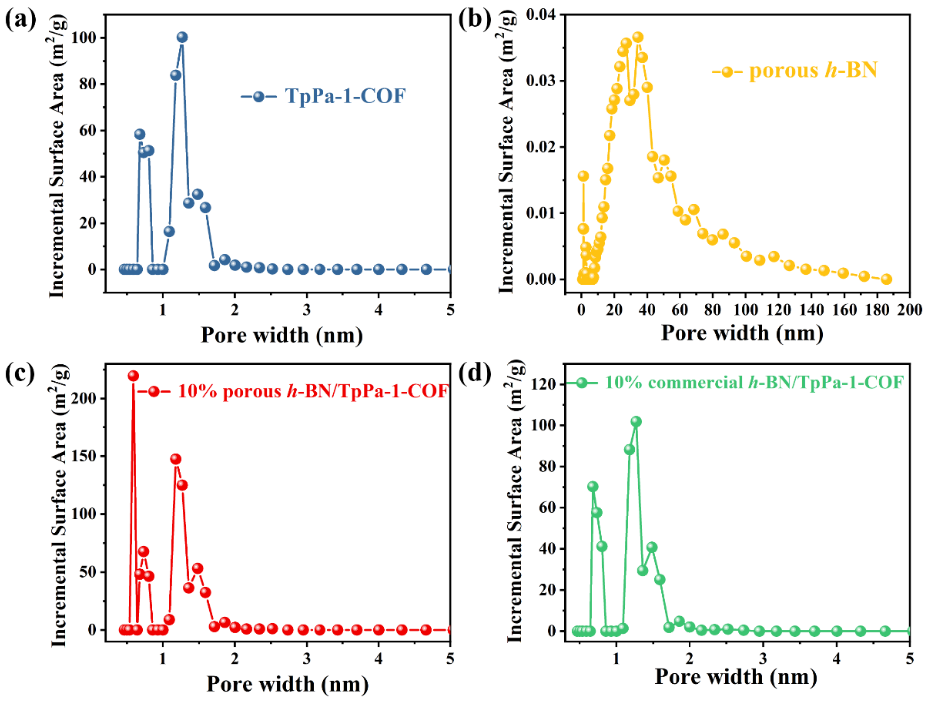

Fig. S7 (a-d) The pore size distribution plots of TpPa-1-COF, porous *h*-BN, 10% porous *h*-BN/TpPa-1-COF and 10% commercial *h*-BN/TpPa-1-COF. The pore size of TpPa-1-COF is approximate 1.2 nm, which is in accordance with their crystal structure. And

porous *h*-BN has mesoporous pores, in which the pore size is estimated to be around 30 nm. Moreover, the pore size distribution of porous *h*-BN/TpPa-1-COF illustrates that it has abundant microporous channels at about 0.1 nm. The larger specific surface area of porous *h*-BN/TpPa-1-COF makes the enrichment of active sites more favorable.

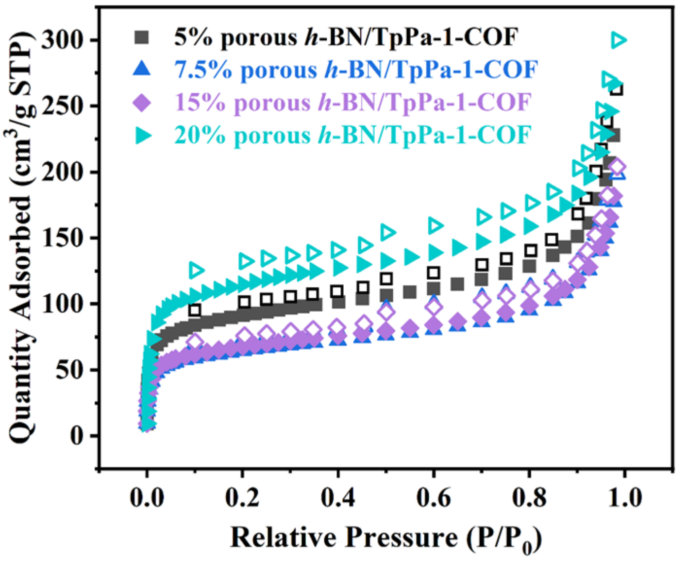

Fig. S8 N_2 adsorption-desorption isotherms of 5% porous *h*-BN/TpPa-1-COF, 7.5% porous *h*-BN/TpPa-1-COF, 15% porous *h*-BN/TpPa-1-COF and 20% porous *h*-BN/TpPa-1-COF

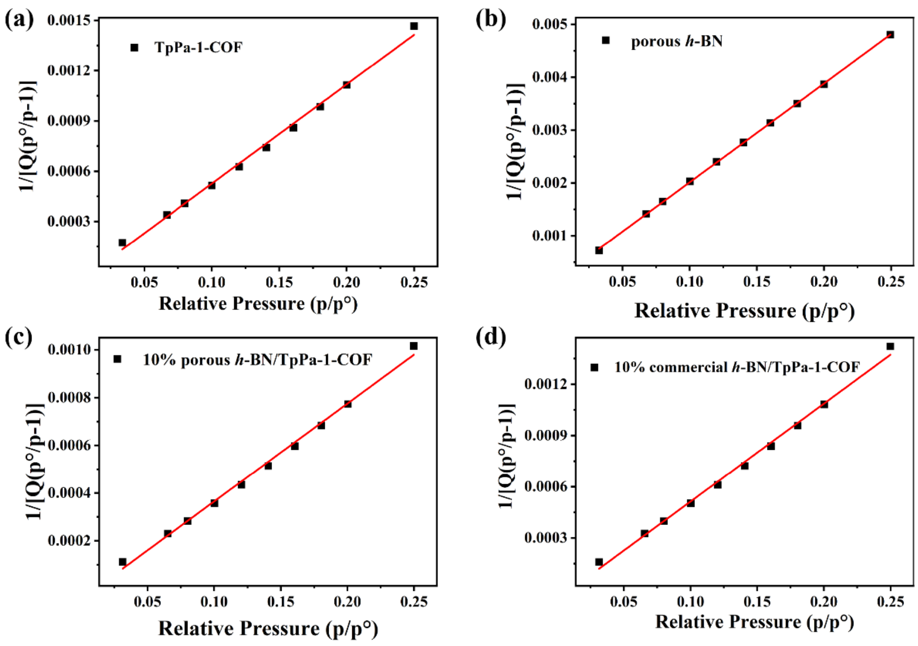

Fig. S9 (a-d) The linear fitting curve for calculating BET surface areas of TpPa-1-COF, porous *h*-BN, 10% porous *h*-BN/TpPa-1-COF and 10% commercial *h*-BN/TpPa-1-COF

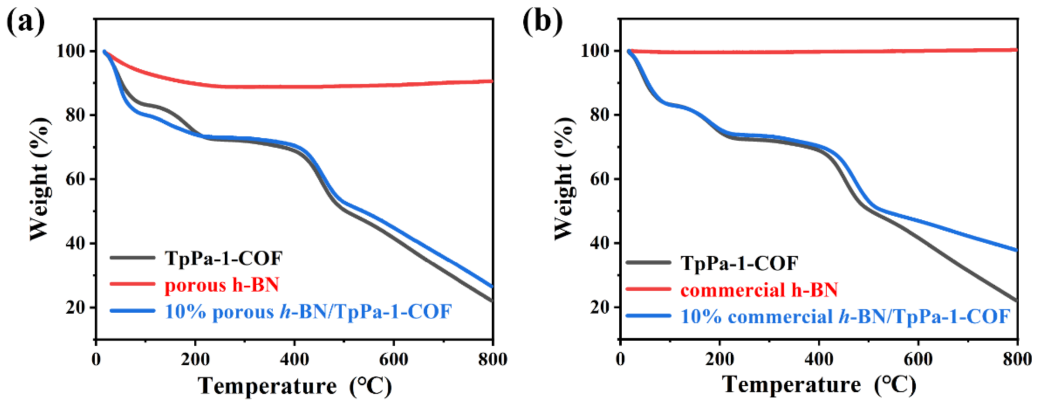

Fig. S10 Thermogravimetric curves of porous *h*-BN, commercial *h*-BN, 10% porous *h*-BN/TpPa-1-COF and 10% commercial *h*-BN/TpPa-1-COF. The decomposition temperature of TpPa-1-COF is about 425 ºC, reveals a relative high thermal stability. After combining two materials, the decomposition temperature of composite is basically consistent with the pristine TpPa-1-COF, which prove that the addition of porous *h*-BN will not break the framework of TpPa-1-COF. Moreover, based on the weight loss, the proportion of two materials is able to be ascertained.

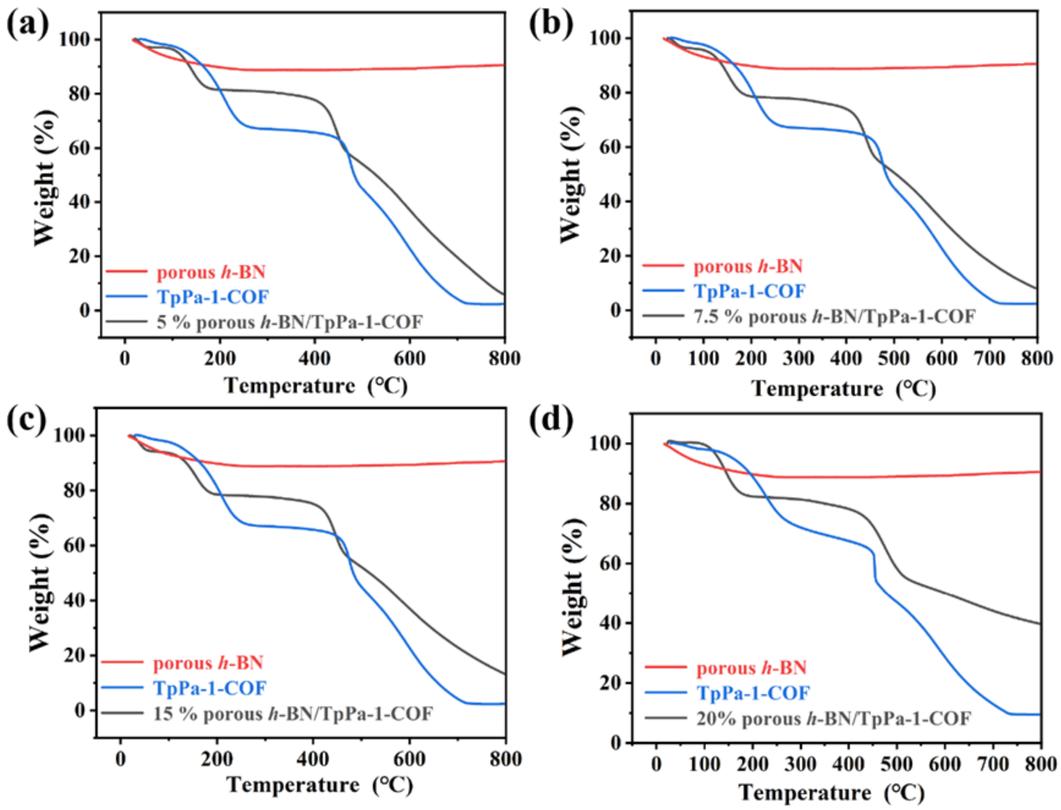

Fig. S11 Thermogravimetric analysis (TGA) curves of 5% porous *h*-BN/TpPa-1-COF, 7.5% porous *h*-BN/TpPa-1-COF, 15% porous *h*-BN/TpPa-1-COF and 20% porous *h*-BN/TpPa-1-COF

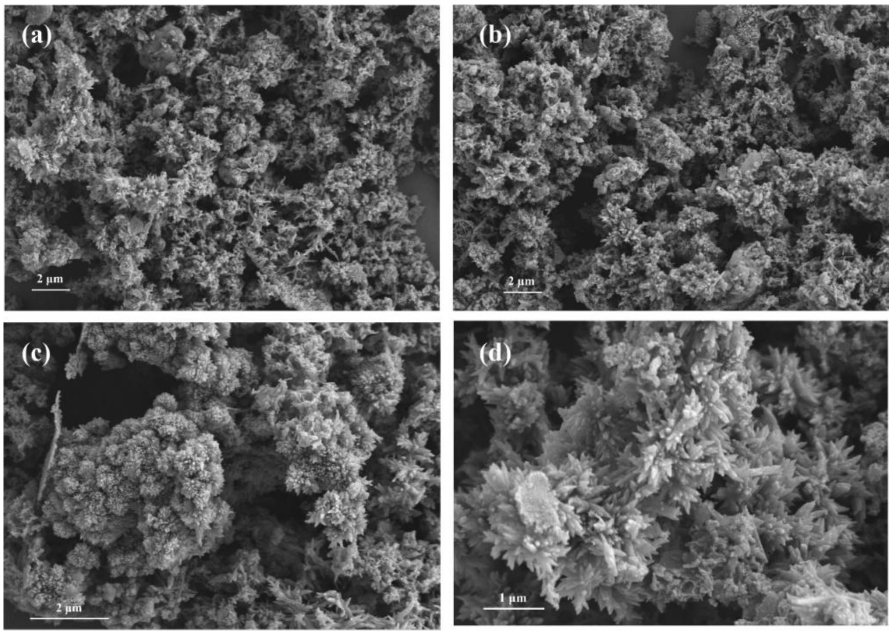

Fig. S12 (**a-d**) SEM of 5% porous *h*-BN/TpPa-1-COF, 7.5% porous *h*-BN/TpPa-1-COF, 15% porous *h*-BN/TpPa-1-COF and 20% porous *h*-BN/TpPa-1-COF

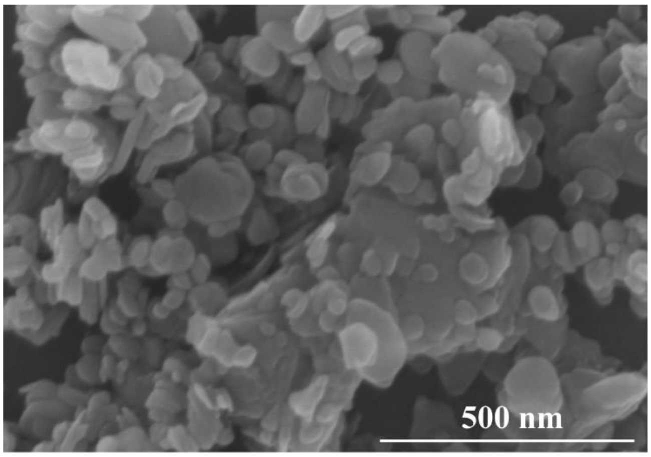

Fig. S13 SEM images of commercial *h*-BN

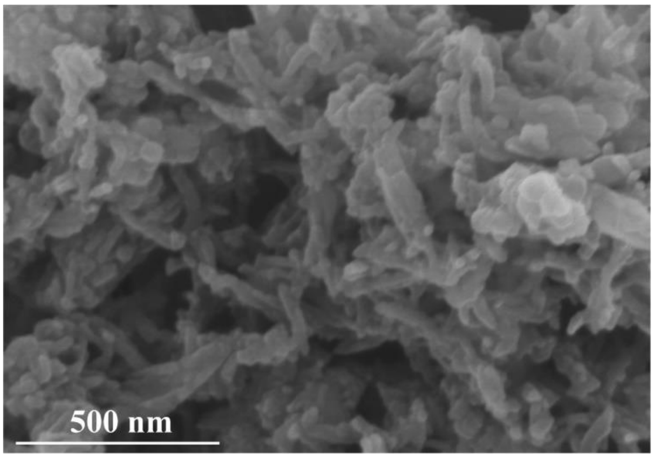

Fig. S14 SEM images of 10% commercial *h*-BN/TpPa-1-COF

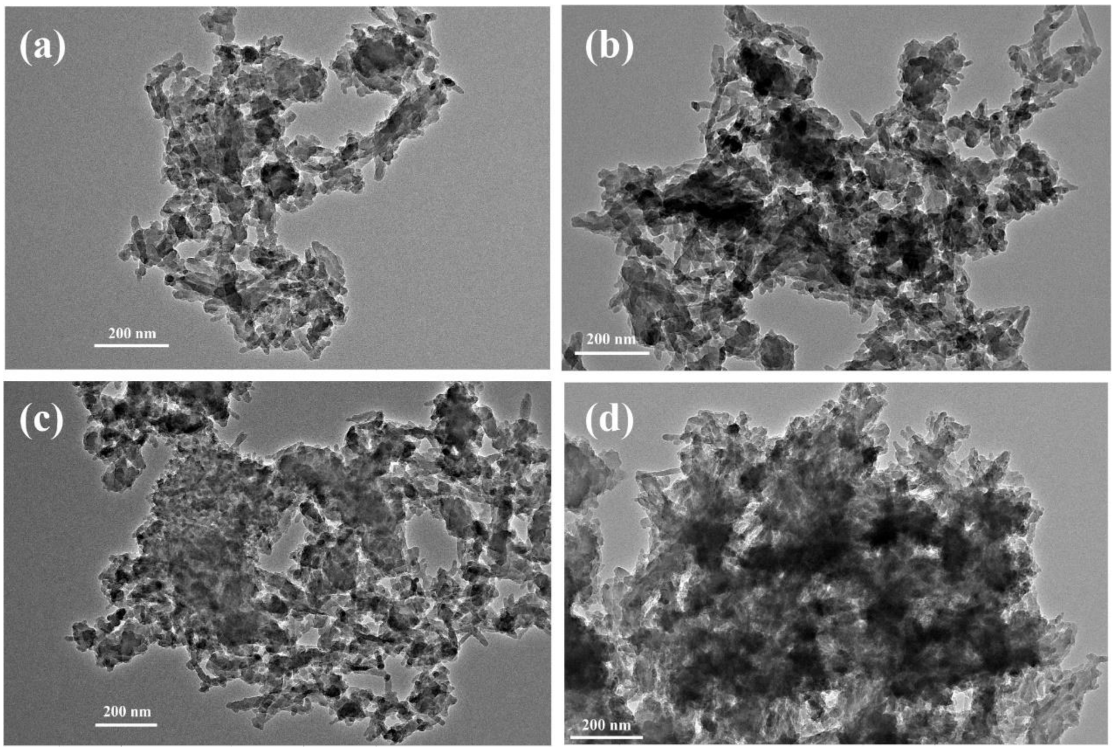

Fig. S15 (**a-d**) TEM of 5% porous h-BN/TpPa-1-COF, 7.5% porous h-BN/TpPa-1-COF, 15% porous h-BN/TpPa-1-COF and 20% porous h-BN/TpPa-1-COF

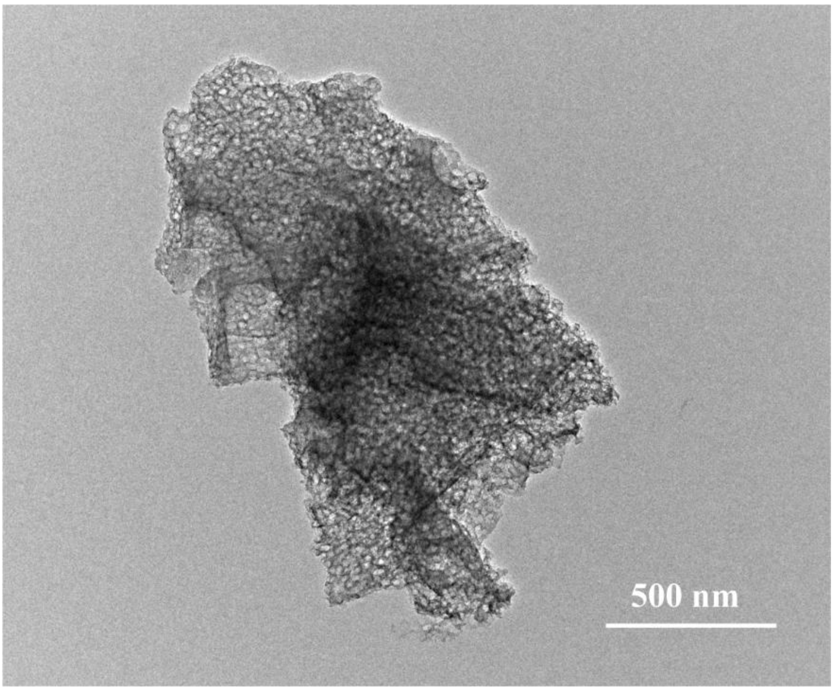

Fig. S16 TEM images of porous *h*-BN

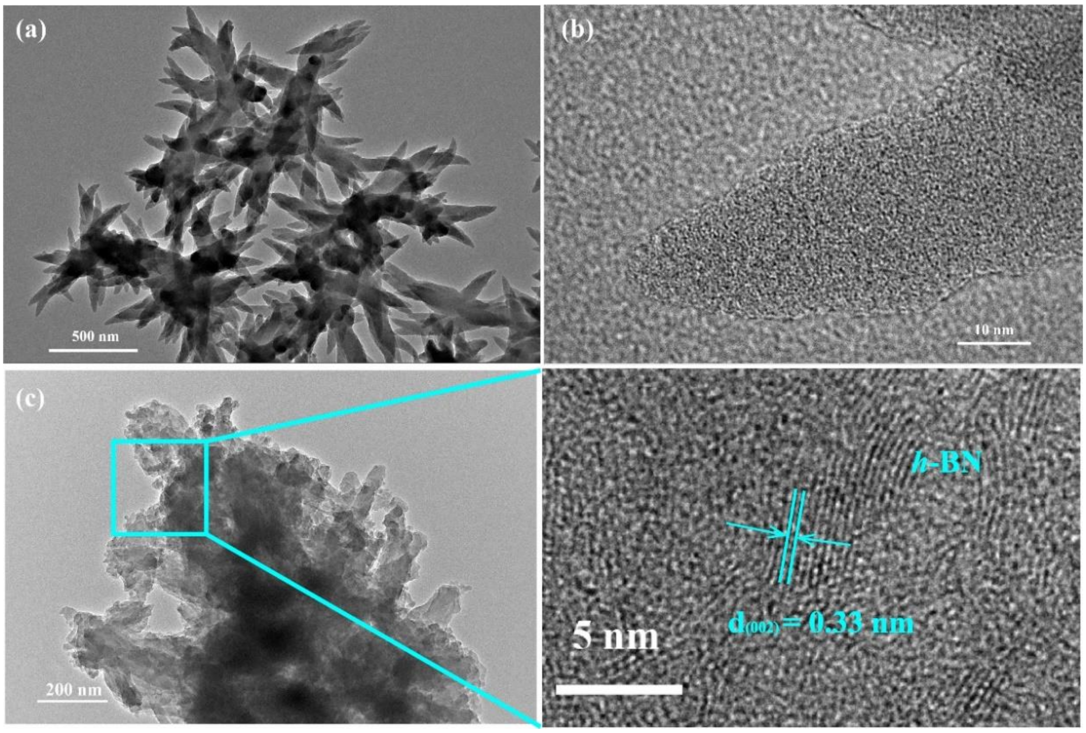

Fig. S17 (**a, c**) TEM images for TpPa-1-COF and 10% porous *h*-BN/TpPa-1-COF and the insert images of (**b, d**) show the HRTEM of 10% TpPa-1-COF and porous h-BN/TpPa-1-COF

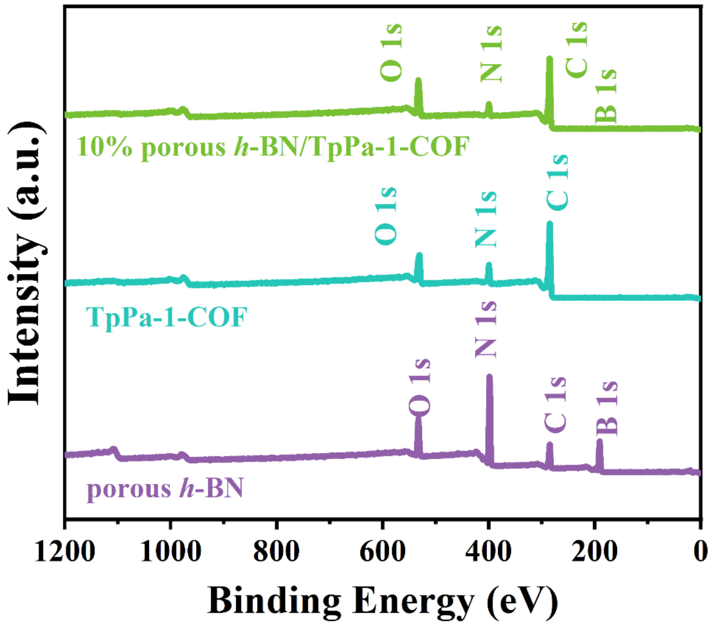

Fig. S18 XPS survey spectra for 10% porous *h*-BN/TpPa-1-COF, TpPa-1-COF and porous *h*-BN. It can be observed that 10% porous *h*-BN/TpPa-1-COF contains C, N, B and O elements without extra elements, which is consistent with EDS mapping results

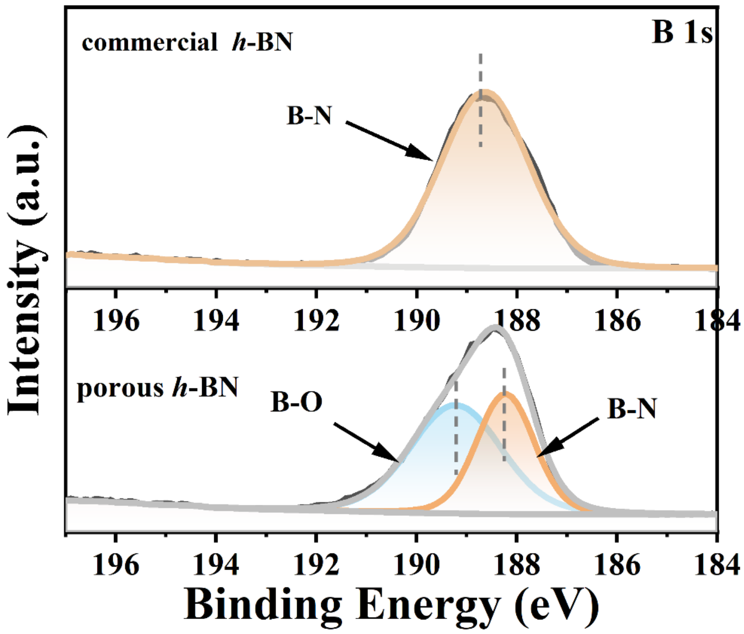

Fig. S19 High-resolution B 1s XPS spectra for commercial *h*-BN and porous *h*-BN

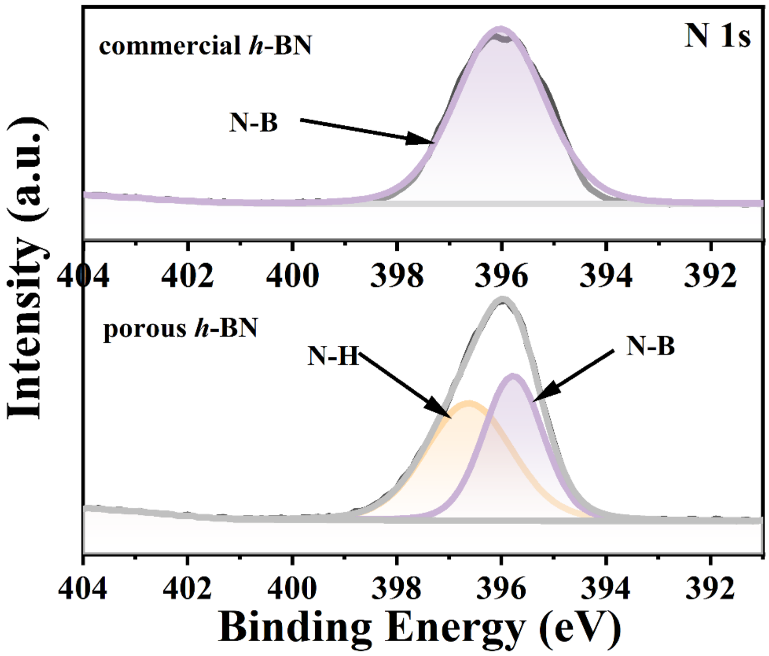

Fig. S20 High-resolution N 1s XPS spectra for commercial *h*-BN and porous *h*-BN

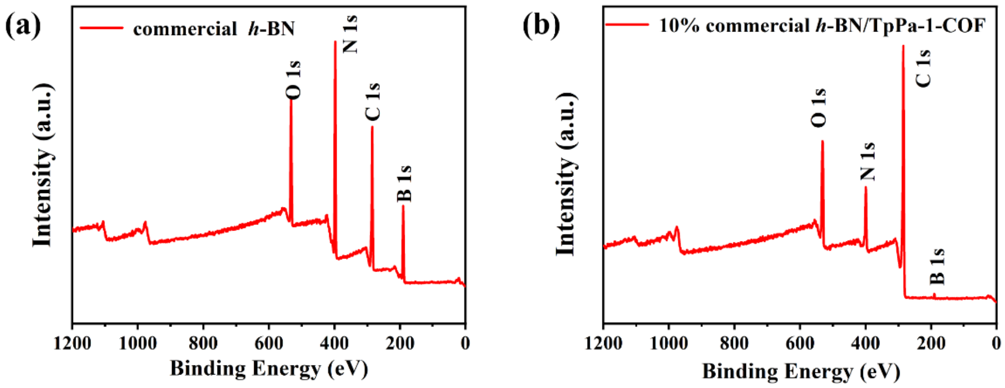

Fig. S21 The full spectrum for 10% commercial *h*-BN/TpPa-1-COF and commercial *h*-BN

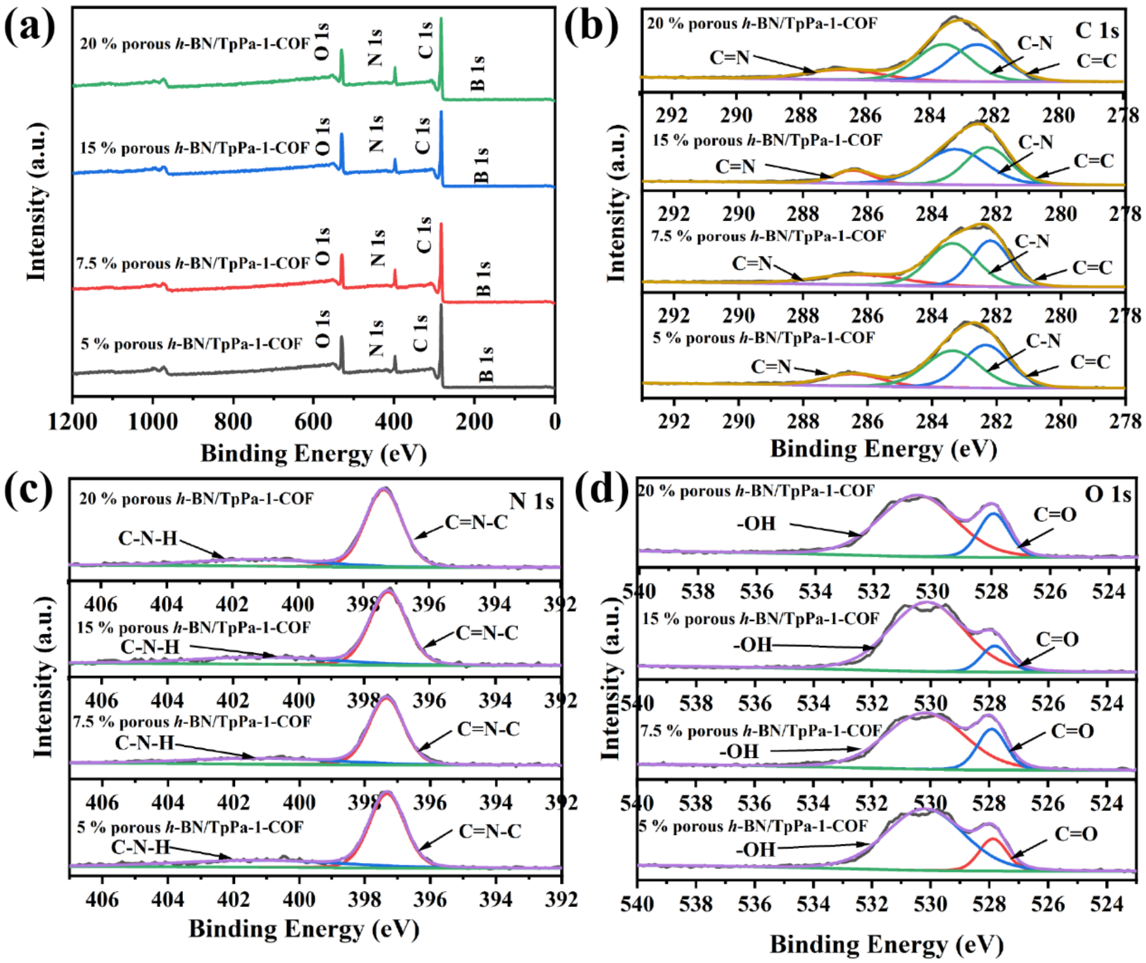

Fig. S22 (**a**) The XPS survey spectra and (**b-d**) High-resolution spectra of 5% porous *h*-BN/TpPa-1-COF, 7.5% porous *h*-BN/TpPa-1-COF, 15% porous *h*-BN/TpPa-1-COF and 20% porous *h*-BN/TpPa-1-COF

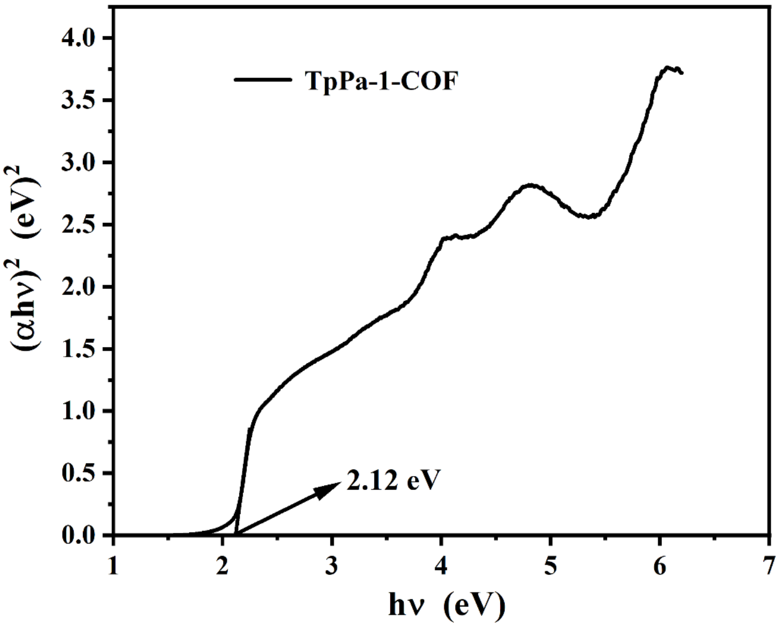

Fig. S23 Tauc plot of TpPa-1-COF. Based on the Tauc diagram of Kubelka-Munk equation ($\alpha h\nu = A(h\nu\text{-}Eg)^2$), the band gap of TpPa-1-COF was calculated to be 2.12 eV

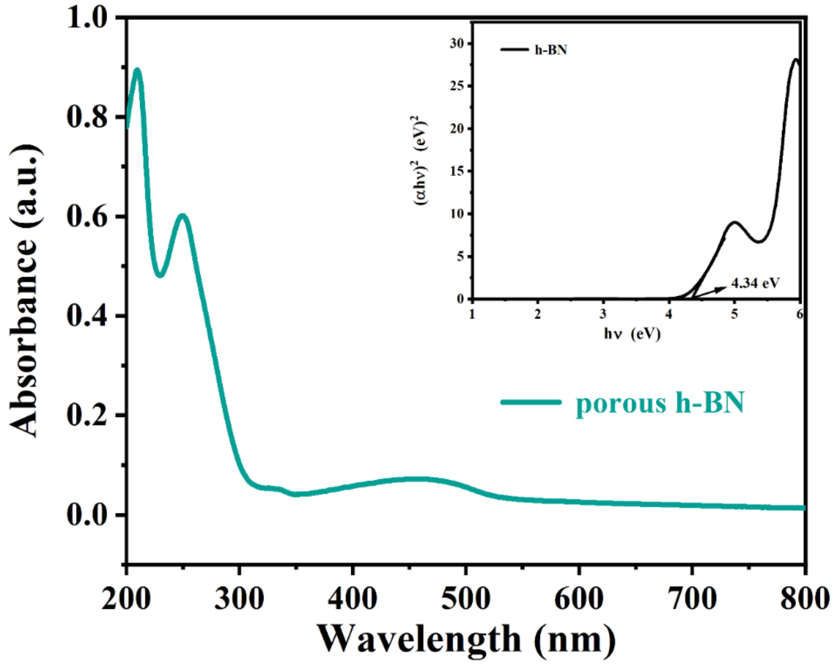

Fig. S24 UV–vis diffuse reflectance spectrum and Tauc plot of porous *h*-BN

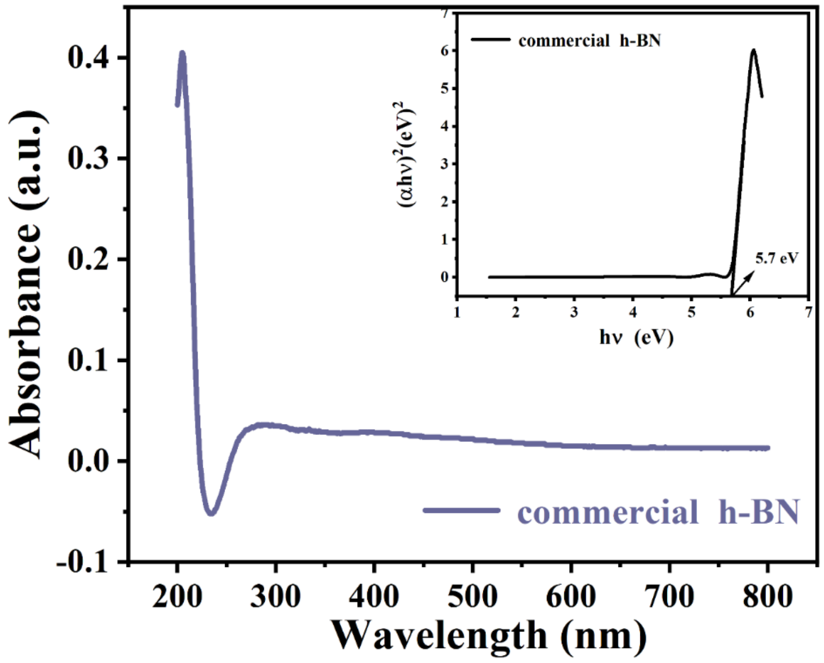

Fig. S25 UV–vis diffuse reflectance spectrum and Tauc plot of commercial *h*-BN

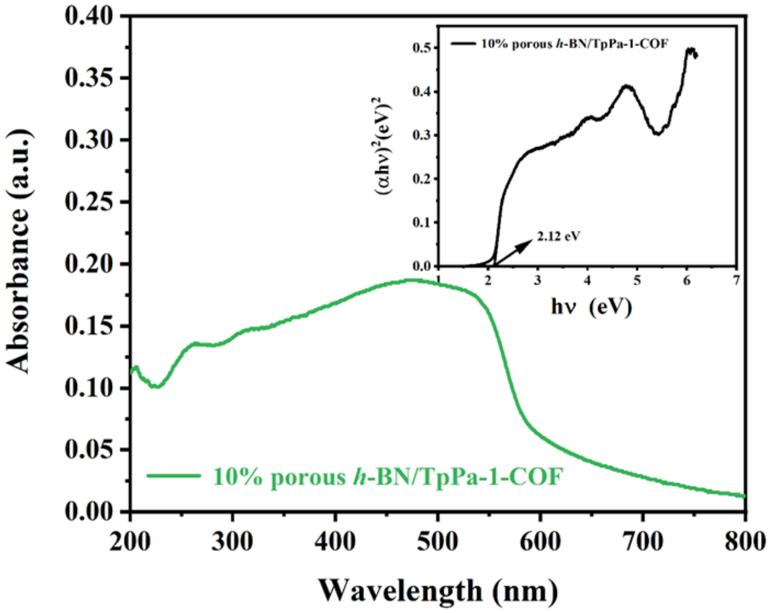

Fig. S26 UV–vis diffuse reflectance spectrum and Tauc plot of 10% porous *h*-BN/TpPa-1-COF

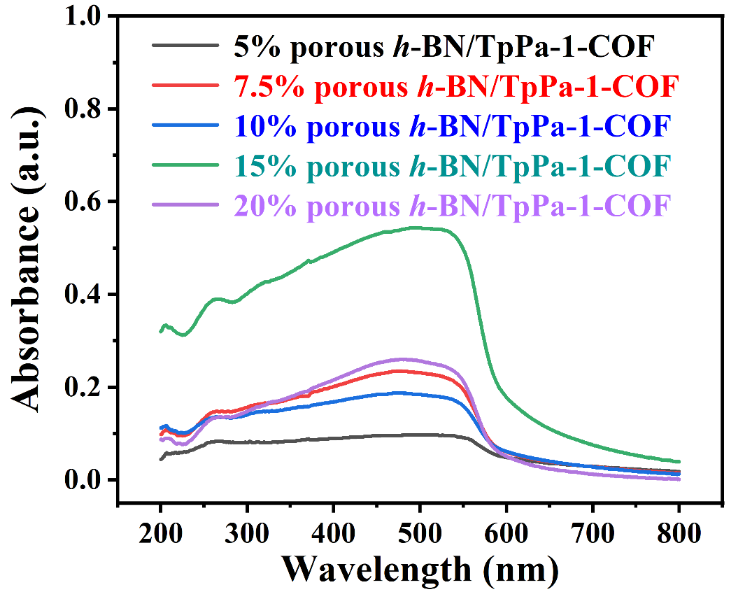

Fig. S27 UV-vis diffuse reflectance spectra of TpPa-1-COF and various ratios of porous *h*-BN/TpPa-1-COF

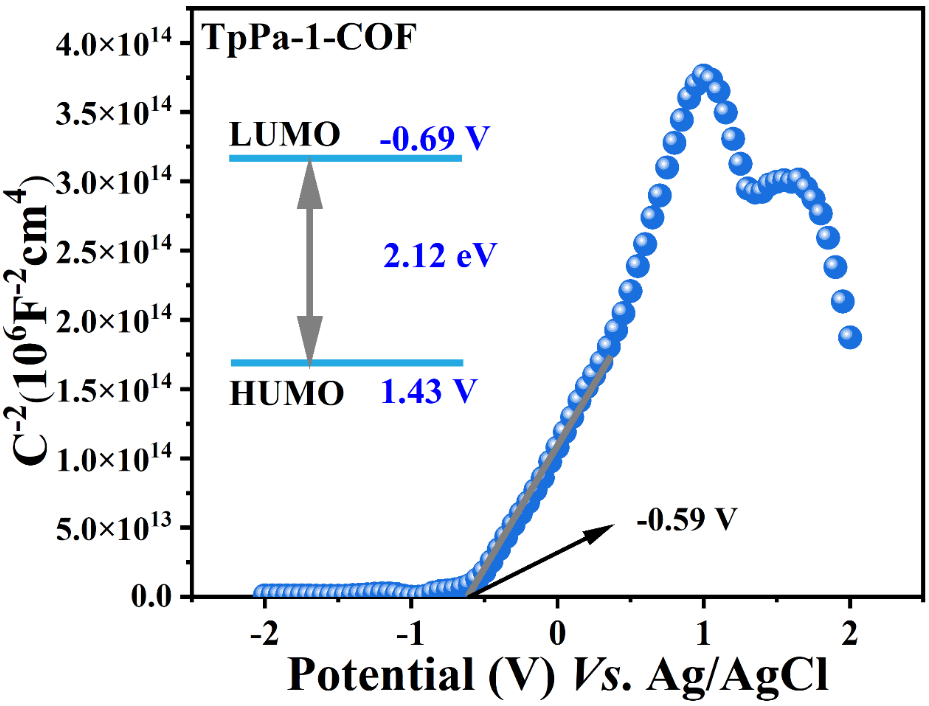

Fig. S28 Mott–Schottky plots for TpPa-1-COF. Mott-Schottky tests experimental results showed that the Fermi energy level of TpPa-1-COF is -0.59 V vs Ag/AgCl

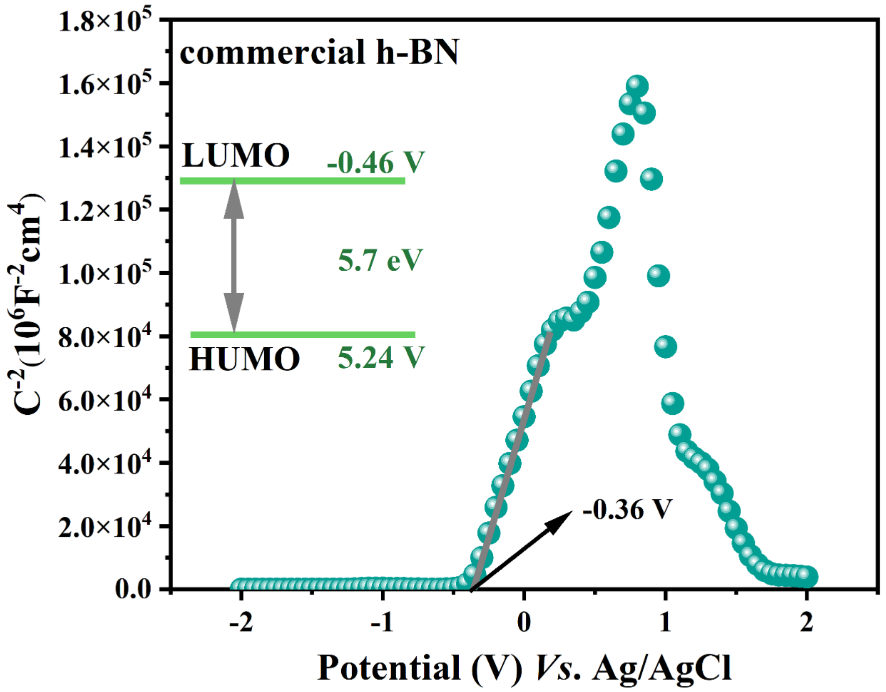

Fig. S29 Mott–Schottky plots for commercial *h*-BN. Mott-Schottky tests experimental results showed that the Fermi energy level of commercial *h*-BN is -0.36 V vs Ag/AgCl

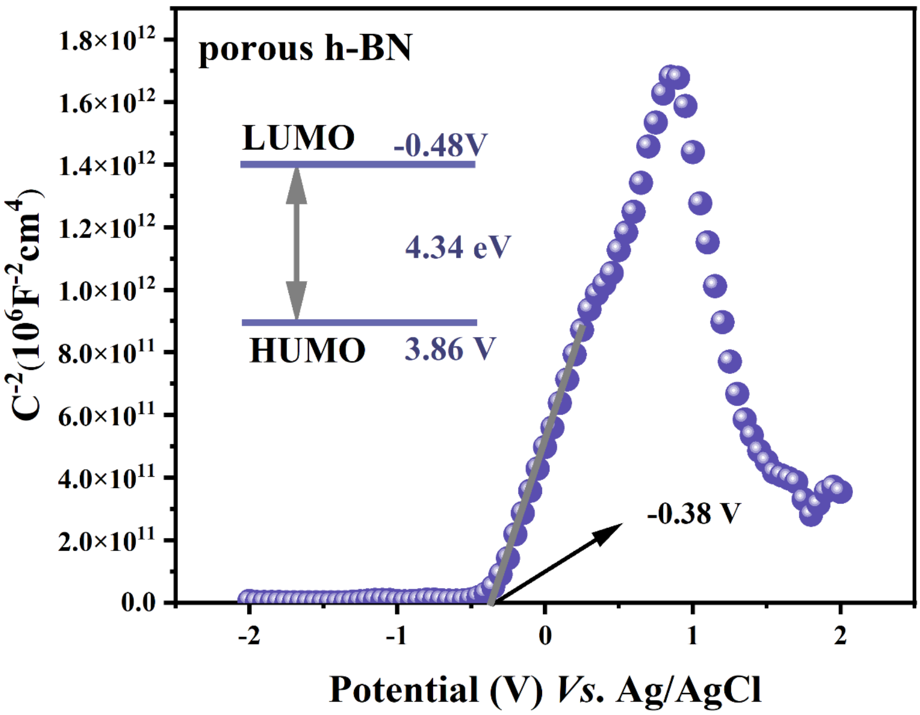

Fig. S30 Mott–Schottky plots for porous *h*-BN. Mott-Schottky tests experimental results showed that the Fermi energy level of porous *h*-BN is -0.38 V vs Ag/AgCl

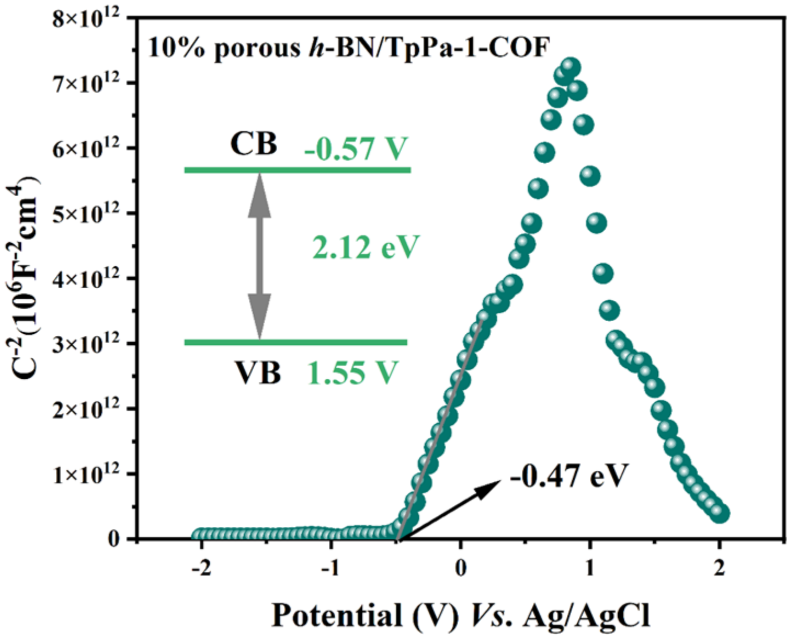

Fig. S31 Mott–Schottky plots for 10% porous *h*-BN/TpPa-1-COF

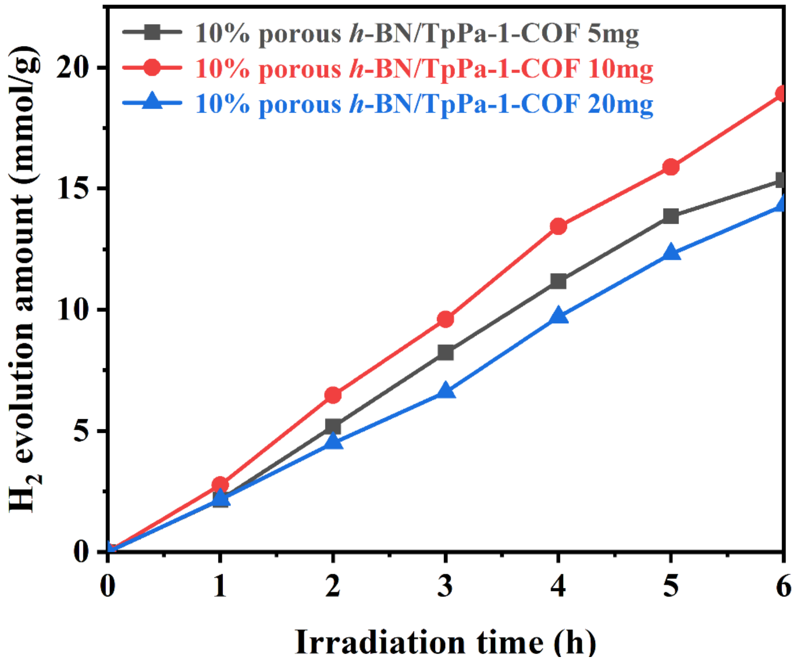

Fig. S32 Comparison of the photocatalytic capacity for 10% porous h-BN/TpPa-1-COF with different mass of added amount

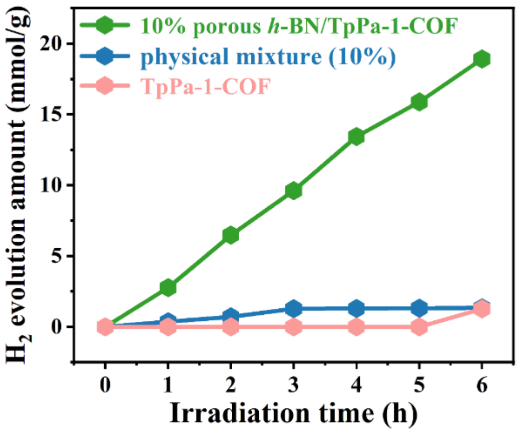

Fig. S33 Comparison of the photocatalytic capacity for 10% porous *h*-BN/TpPa-1-COF, physically mixed (10%) hybrid, TpPa-1-COF

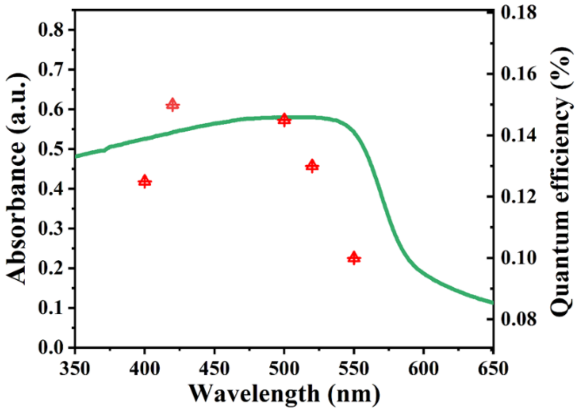

Fig. S34 Wavelength-dependent apparent quantum efficiency (AQE) of 10% porous *h*-BN/TpPa-1-COF in 5 mg

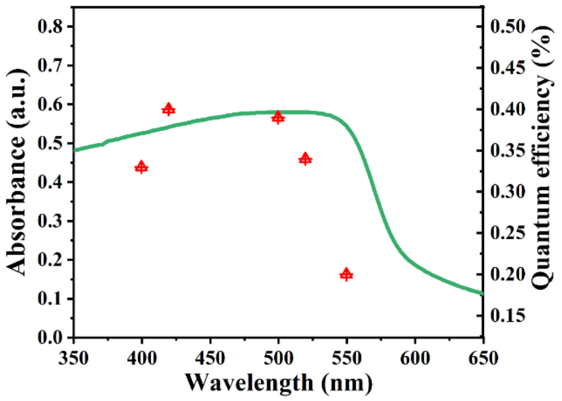

Fig. S35 Wavelength-dependent apparent quantum efficiency (AQE) of 10% porous *h*-BN/TpPa-1-COF in 10 mg

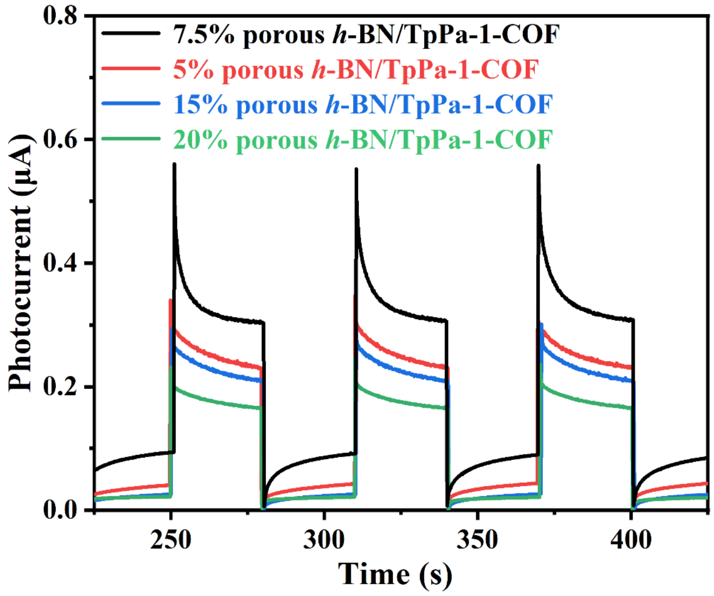

Fig. S36 Transient photocurrent response 5% porous *h*-BN/TpPa-1-COF, 7.5% porous *h*-BN/TpPa-1-COF, 15% porous *h*-BN/TpPa-1-COF and 20% porous *h*-BN/TpPa-1-COF

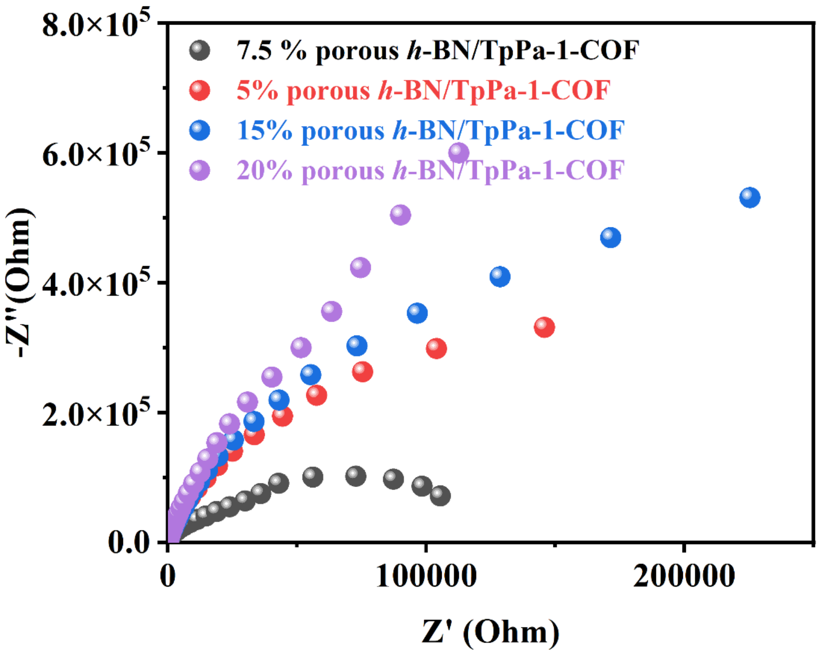

Fig. S37 EIS Nyquist plots of 5% porous *h*-BN/TpPa-1-COF, 7.5% porous *h*-BN/TpPa-1-COF, 15% porous *h*-BN/TpPa-1-COF and 20% porous *h*-BN/TpPa-1-COF

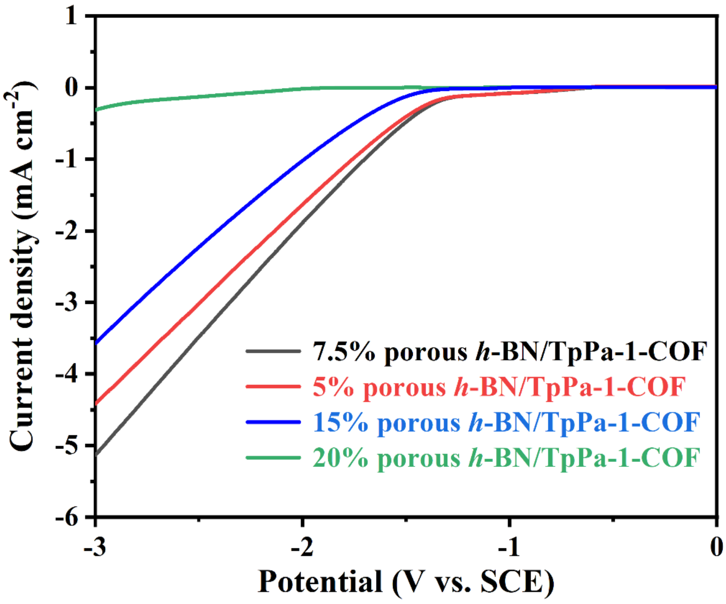

Fig. S38 LSV curves of 5% porous *h*-BN/TpPa-1-COF, 7.5% porous *h*-BN/TpPa-1-COF, 15% porous *h*-BN/TpPa-1-COF and 20% porous *h*-BN/TpPa-1-COF

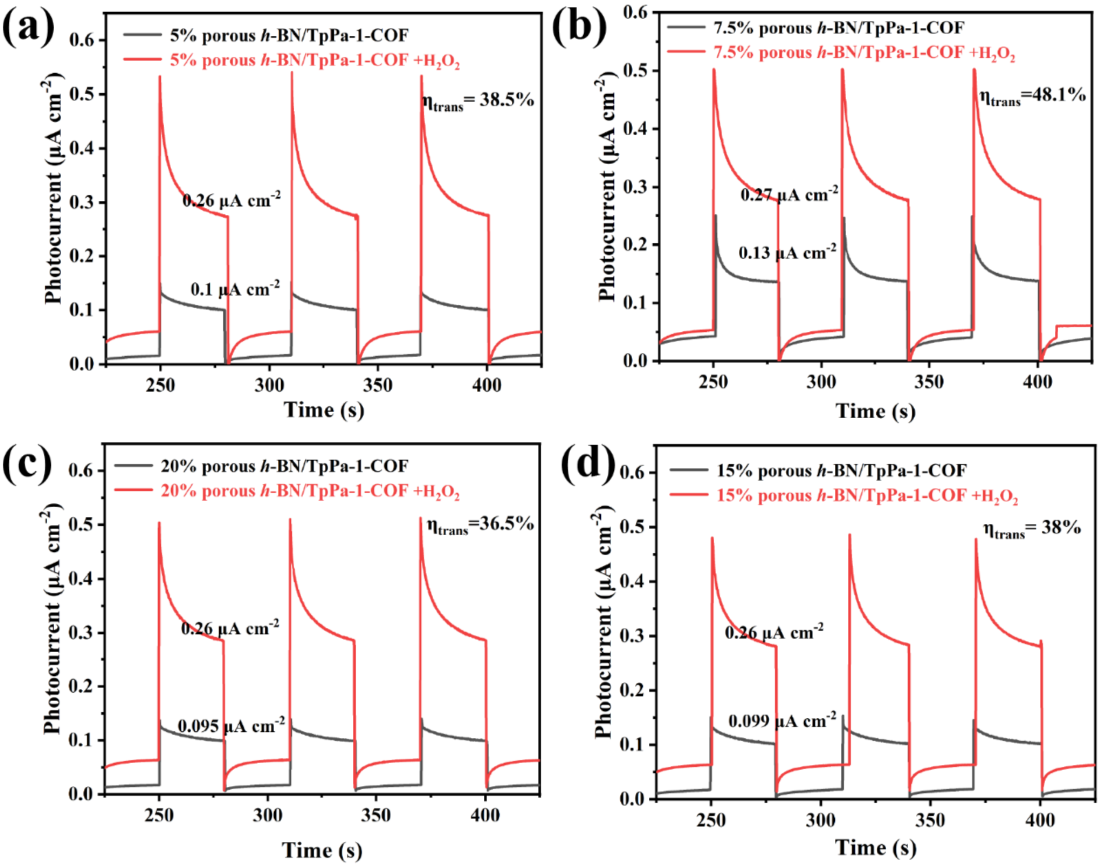

Fig. S39 (**a-d**) The photocurrent response of without and with adding H_2O_2 into electrolyte of 5% porous *h*-BN/TpPa-1-COF, 7.5% porous *h*-BN/TpPa-1-COF, 15% porous *h*-BN/TpPa-1-COF and 20% porous *h*-BN/TpPa-1-COF

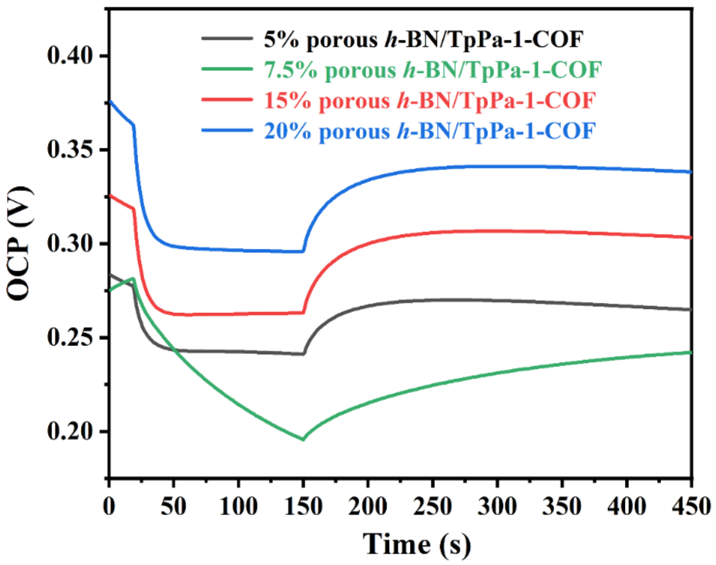

Fig. S40 OCVD curves of 5% porous *h*-BN/TpPa-1-COF, 7.5% porous *h*-BN/TpPa-1-COF, 15% porous *h*-BN/TpPa-1-COF and 20% porous *h*-BN/TpPa-1-COF

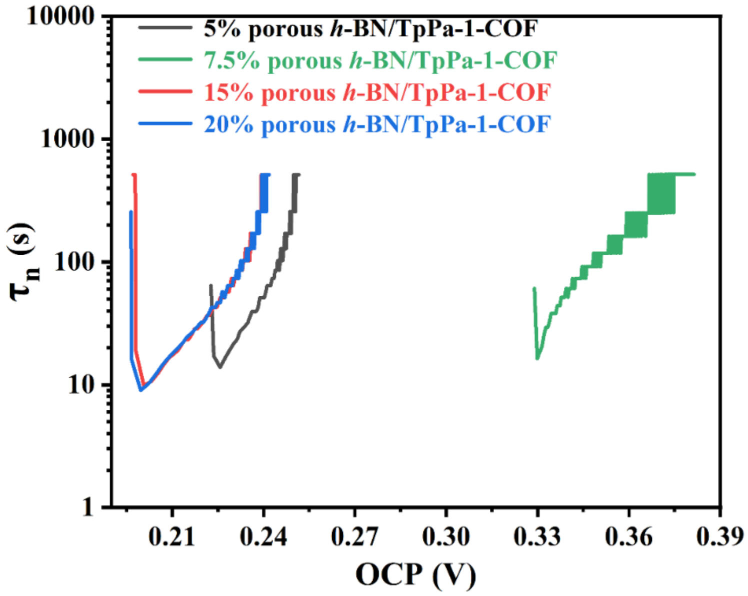

Fig. S41 The average lifetime of the photogenerated carriers (τn) for 5% porous *h*-BN/TpPa-1-COF, 7.5% porous *h*-BN/TpPa-1-COF, 15% porous *h*-BN/TpPa-1-COF and 20% porous *h*-BN/TpPa-1-COF

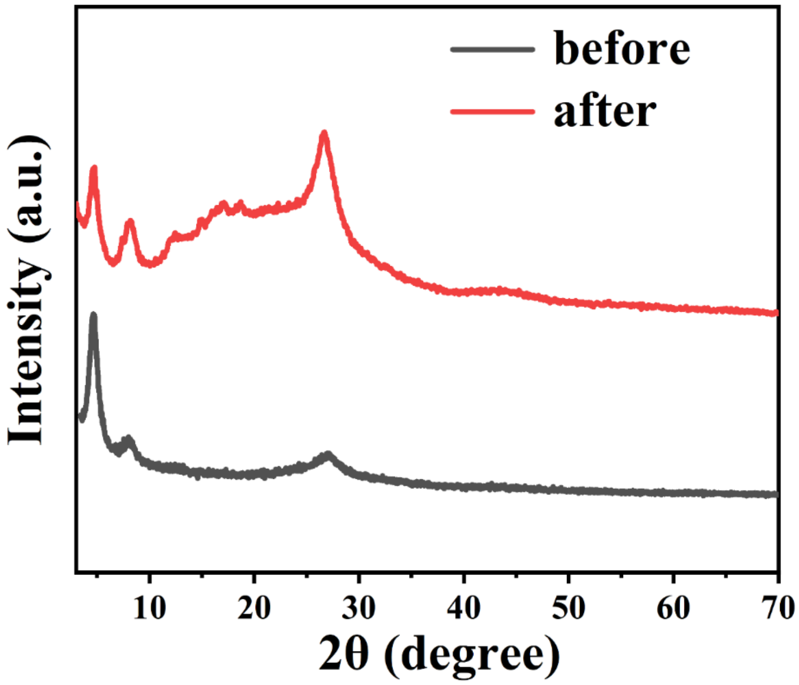

Fig. S42 XRD patterns of 10% porous *h*-BN/TpPa-1-COF before and after photocatalytic reaction. After the photocatalytic hydrogen production reaction, the diffraction peaks of 10% porous *h*-BN/TpPa-1-COF at 4.6° and 26° are still present, indicating that the structure is well maintained

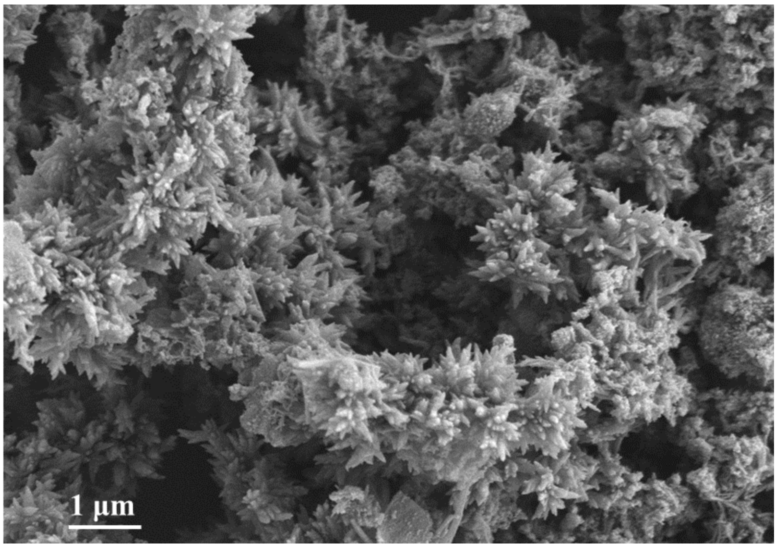

Fig. S43 SEM image of 10% porous *h*-BN/TpPa-1-COF after photocatalytic reaction. The SEM image of 10% porous *h*-BN/TpPa-1-COF in the recycling hydrogen production experiment still clearly shown the nano-flower-like structure of TpPa-1-COF and the layered porous *h*-BN bound to TpPa-1-COF

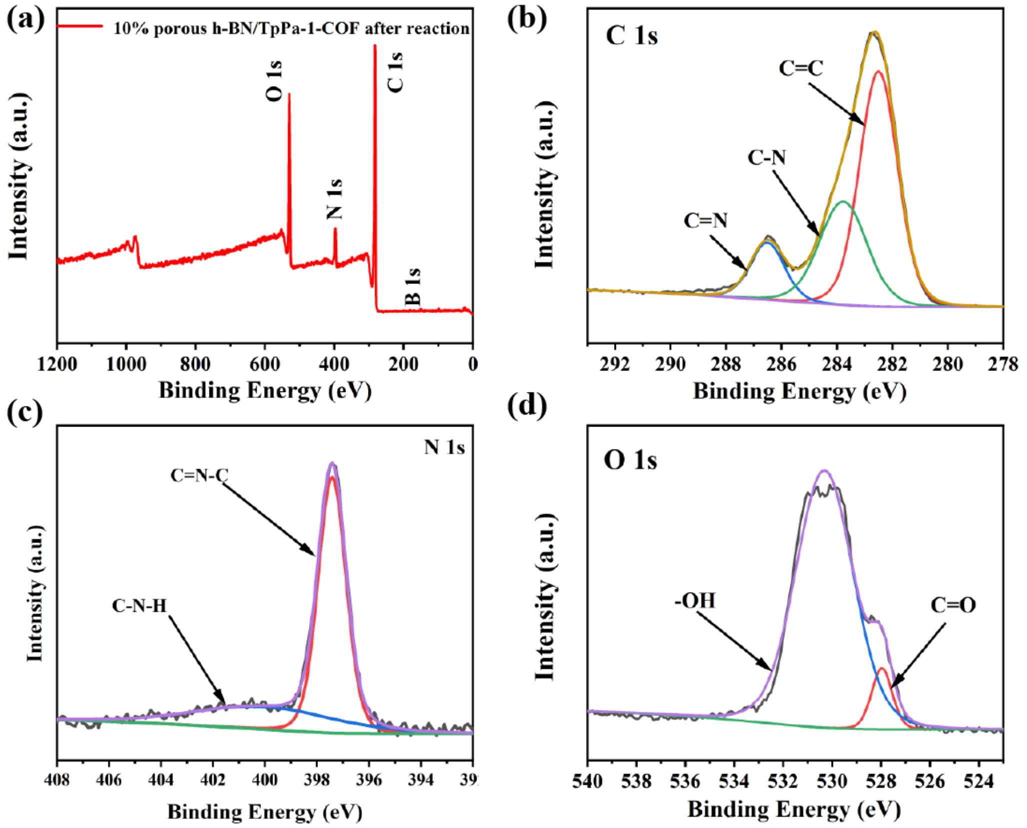

Fig. S44 (**a**) The XPS survey spectra and High-resolution spectra of (**b**) C 1s, (**c**) N 1s, (**d**) O 1s for 10% porous *h*-BN/TpPa-1-COF after photocatalytic reaction. The 10% porous *h*-BN/TpPa-1-COF elements stay the same after the photocatalytic reaction, and the bonding in the complex remains essentially unchanged

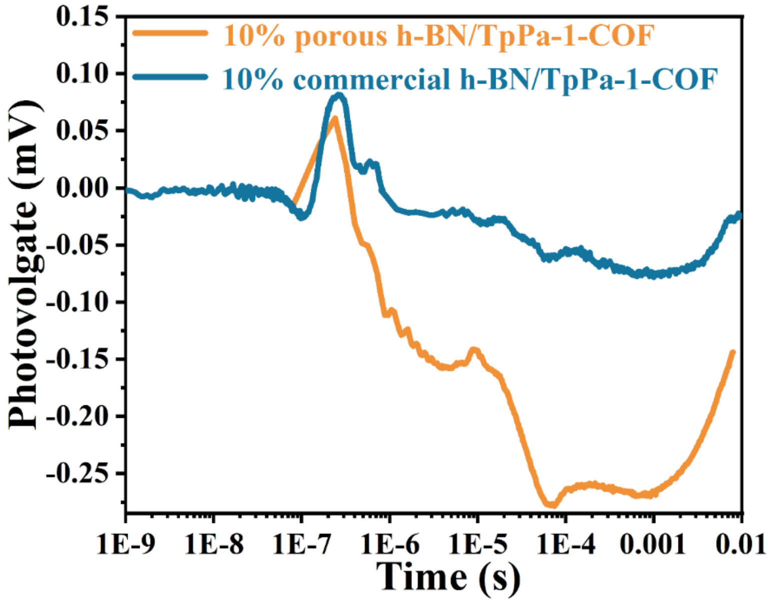

Fig. S45 TPV spectra of 10% commercial *h*-BN/TpPa-1-COF and 10% porous *h*-BN/TpPa-1-COF

Table S1 Summary of H_2 evolution activity of metal-free photocatalyts

Catalyst	Cocatalyst	Illumination	Activity, μmol·g^{-1}·h^{-1}	AQE	Refs.
10% porous *h*-BN/TpPa-1-COF	-	λ>420nm	3150		This work
10% commercial *h*-BN/TpPa-1-COF	-	λ>420nm	1820		This work
BP/g-C_3N_4	-	λ>420nm	384.17	-	S17
BP-BM	-	λ>420nm	512	0.47% λ=420±5 nm	S18
bulk BP	-	λ>420nm	28	0.0008% λ=420±5 nm	S18
BP/CN	-	λ>420nm	786	-	S19
3D N-doped	-	λ>420nm	480	27.8% λ=420±15nm	S20
C-I codoped	-	λ>420nm	168	-	S21
CCN-50	Pt	λ>420nm	746.95	-	S22
g-C_3N_4-1	Pt	λ>420nm	258.5	-	S23
CTF-1/rGO-2	Pt	λ>420nm	894	6.4% λ=420nm	S24
$C_{0.24}$/CTF-1	Pt	λ>420nm	2240	0.73% λ=400nm	S25
NP-CN24	Pt	λ>420nm	851.9	-	S26
CTFCl	Pt	λ>420nm	1400	10.31% λ=420nm	S27
CTFBr	Pt	λ>420nm	1315	-	S27
CTFF	Pt	λ>420nm	787.5	-	S27
15N-CNU	Pt	λ>420nm	2180	-	S28
TFPT-COF	Pt	λ>420nm	1970	2.2% λ=400±20nm	S29
PMDA-COF	Pt	λ>420nm	435.6	-	S30
TP-BDDA COF	Pt	λ>395nm	324	-	S31
PyG-Im-COF	Pt	λ>420nm	1866	0.39% λ=420nm	S32
PyG-COF	Pt	λ>420nm	654	-	S32
TAB-TFP-COF	Pt	λ>420nm	1140	0.69% λ=420nm	S33

Supplementary References

[S1] Z. He, C. Kim, L. Lin, T. H. Jeon, S. Lin et al., Formation of heterostructures via direct growth CN on h-BN porous nanosheets for metal-free photocatalysis. Nano Energy **42**, 58-68 (2017). https://doi.org/10.1016/j.nanoen.2017.10.043

[S2] M.-Y. Gao, C.-C. Li, H.-L. Tang, X.-J. Sun, H. Dong et al., Boosting visible-light-driven hydrogen evolution of covalent organic frameworks through compositing with MoS_2: A promising candidate for noble-metal-free photocatalysts. J. Mater. Chem. A **7**(35), 20193-20200 (2019). https://doi.org/10.1039/C9TA07319A

[S3] G. Kresse, J. Hafner. Ab initio molecular dynamics for liquid metals. Phys. Rev. B **47**(1), 558-561 (1993). https://doi.org/10.1103/PhysRevB.47.558

[S4] G. Kresse, J. Hafner, Ab initio molecular-dynamics simulation of the liquid-metal--amorphous-semiconductor transition in germanium. Phys. Rev. B **49**(20), 14251-14269 (1994). https://doi.org/10.1103/PhysRevB.49.14251

[S5] G. Kresse, J. Furthmüller, Efficiency of ab-initio total energy calculations for metals and semiconductors using a plane-wave basis set. Comp. Mater. Sci. **6**(1), 15-50 (1996). https://doi.org/10.1016/0927-0256(96)00008-0

[S6] G. Kresse, J. Furthmüller, Efficient iterative schemes for ab initio total-energy calculations using a plane-wave basis set. Phys. Rev. B **54**(16), 11169-11186 (1996). https://doi.org/10.1103/PhysRevB.54.11169

[S7] J. P. Perdew, M. Ernzerhof, K. Burke, Rationale for mixing exact exchange with density functional approximations. J. Chem. Phys. **105**(22), 9982-9985 (1996). https://doi.org/10.1063/1.472933

[S8] P. E. Blöchl, Projector augmented-wave method. Phys. Rev. B **50**(24), 17953-17979 (1994). https://doi.org/10.1103/PhysRevB.50.17953

[S9] S. L. Dudarev, G. A. Botton, S. Y. Savrasov, C. J. Humphreys, A. P. Sutton, Electron-energy-loss spectra and the structural stability of nickel oxide: An LSDA+U study. Phys. Rev. B. **57**(3), 1505-1509 (1998). https://doi.org/10.1103/PhysRevB.57.1505

[S10] L. Wang, T. Maxisch, G. Ceder, Oxidation energies of transition metal oxides within the GGA+U framework. Phys. Rev. B **73**(19), 195107 (2006). https://doi.org/10.1103/PhysRevB.73.195107

[S11] M. K. Aydinol, A. F. Kohan, G. Ceder, K. Cho, J. Joannopoulos, Ab initio study of lithium intercalation in metal oxides and metal dichalcogenides. Phys. Rev. B **56**(3), 1354-1365 (1997). https://doi.org/10.1103/PhysRevB.56.1354

[S12] Z. Zhao, Z. Yang, Y. Wen, Y. Wang, Facile synthesis and characterization of hexagonal boron nitride nanoplates by two-step route. J. Am. Ceram. Soc. **94**, 4496-4501 (2011). https://doi.org/https://doi.org/10.1111/j.1551-2916.2011.04752.x

[S13] J. Yu, L. Qin, Y. Hao, S. Kuang, X. Bai et al., Vertically aligned boron nitride nanosheets: Chemical vapor synthesis, ultraviolet light emission, and superhydrophobicity. ACS Nano **4**, 414-422 (2010). https://doi.org/10.1021/nn901204c

[S14] D. M. Hoffman, G. L. Doll, P. C. Eklund, Optical properties of pyrolytic boron nitride in the energy range 0.05---10 ev. Phys. Rev. B **30**, 6051-6056 (1984). https://doi.org/10.1103/PhysRevB.30.6051

[S15] R. Geick, C. H. Perry, G. Rupprecht, Normal modes in hexagonal boron nitride. Phys. Rev. **146**, 543-547 (1966). https://doi.org/10.1103/PhysRev.146.543

[S16] R. J. Nemanich, S. A. Solin, R. M. Martin, Light scattering study of boron nitride microcrystals. Phys. Rev. B **23**, 6348-6356 (1981). https://doi.org/10.1103/PhysRevB.23.6348

[S17] Q. Zhang, S. Huang, J. Deng, D. T. Gangadharan, F. Yang et al., Ice-assisted synthesis of black phosphorus nanosheets as a metal-free photocatalyst: 2D/2D heterostructure for broadband H_2 evolution. Adv. Funct. Mater. **29**, 1902486 (2019). https://doi.org/10.1002/adfm.201902486

[S18] X. Zhu, T. Zhang, Z. Sun, H. Chen, J. Guan et al., Black phosphorus revisited: A missing metal-free elemental photocatalyst for visible light hydrogen evolution. Adv. Mater. **29**, 1605776 (2017). https://doi.org/10.1002/adma.201605776

[S19] M. Wen, J. Wang, R. Tong, D. Liu, H. Huang et al., A low-cost metal-free photocatalyst based on black phosphorus. Adv. Sci. **6**, 1801321 (2019). https://doi.org/10.1002/advs.201801321

[S20] N. Tian, Y. Zhang, X. Li, K. Xiao, X. Du et al., Precursor-reforming protocol to 3D mesoporous g-C_3N_4 established by ultrathin self-doped nanosheets for superior hydrogen evolution. Nano Energy **38**, 72-81 (2017). https://doi.org/10.1016/j.nanoen.2017.05.038

[S21] C. Yang, W. Teng, Y. Song, Y. Cui, C-i codoped porous g-C_3N_4 for superior photocatalytic hydrogen evolution. Chin. J. Catal. **39**, 1615-1624 (2018). https://doi.org/10.1016/S1872-2067(18)63131-6

[S22] X. Luo, Z. Wu, Y. Liu, S. Ding, Y. Zheng et al., Engineering amorphous carbon onto ultrathin g-C_3N_4 to suppress intersystem crossing for efficient photocatalytic H_2 evolution. Adv. Mater. Interfaces **5**, 1800859 (2018). https://doi.org/10.1002/admi.201800859

[S23] W. Xing, C. Li, G. Chen, Z. Han, Y. Zhou et al., Incorporating a novel metal-free interlayer into g-C_3N_4 framework for efficiency enhanced photocatalytic H_2 evolution activity. Appl. Catal. B Environ. **203** (2017). https://doi.org/10.1016/j.apcatb.2016.09.075

[S24] Z. Tan, P. Zhang, Q. Chen, S. Fang, G. Huang et al., Visible-light-driven photocatalyst based upon metal-free covalent triazine-based frameworks for enhanced hydrogen production. Catal. Sci. Technol. **11**, 1874-1880 (2021). https://doi.org/10.1039/D0CY02094J

[S25] Y. Chen, G. Huang, Y. Gao, Q. Chen, J. Bi, Up-conversion fluorescent carbon quantum dots decorated covalent triazine frameworks as efficient metal-free

photocatalyst for hydrogen evolution. Int. J. Hydrogen Energy **47**, 8739-8748 (2022). https://doi.org/10.1016/j.ijhydene.2021.12.220

[S26] G. Wu, C. Liu, W. Shao, Y. Wang, Y. Zhang et al., Nitrogen vacancies and metal-free group intercalation on carbon nitride for super photocatalytic performance. Int. J. Hydrogen Energy **47**, 20540-20545 (2022). https://doi.org/10.1016/j.ijhydene.2022.04.117

[S27] Z. Cheng, K. Zheng, G. Lin, S. Fang, L. Li et al., Constructing a novel family of halogen-doped covalent triazine-based frameworks as efficient metal-free photocatalysts for hydrogen production. Nanoscale Adv. **1**, 2674-2680 (2019). https://doi.org/10.1039/C9NA00089E

[S28] J.-P. Zou, L.-C. Wang, J. Luo, Y.-C. Nie, Q.-J. Xing et al., Synthesis and efficient visible light photocatalytic H_2 evolution of a metal-free g-C_3N_4/graphene quantum dots hybrid photocatalyst. Appl. Catal. B Environ. **193**, 103-109 (2016). https://doi.org/10.1016/j.apcatb.2016.04.017

[S29] L. Stegbauer, K. Schwinghammer, B. V. Lotsch, A hydrazone-based covalent organic framework for photocatalytic hydrogen production. Chem. Sci. **5**, 2789-2793 (2014). https://doi.org/10.1039/C4SC00016A

[S30] R. Lu, C. Liu, Y. Chen, L. Tan, G. Yuan et al., Effect of linkages on photocatalytic H2 evolution over covalent organic frameworks. J. Photochem. Photobiol. A Chem. 421 (2021). https://doi.org/10.1016/j.jphotochem.2021.113546

[S31] P. Pachfule, A. Acharjya, J. Roeser, T. Langenhahn, M. Schwarze et al., Diacetylene functionalized covalent organic framework (COF) for photocatalytic hydrogen generation. J. Am. Chem. Soc. **140**, 1423-1427 (2018). https://doi.org/10.1021/jacs.7b11255

[S32] H. Liu, Y. Li, L. Dai, X. Meng, A. Dong et al., Post-cyclization of a bisimine-linked covalent organic framework to enhance the performance of visible-light photocatalytic hydrogen evolution. Polym. Chem. (2023). https://doi.org/10.1039/D3PY00181D

[S33] X. Wu, M. Zhang, Y. Xia, C. Ru, P. Chen et al., Arylboron functional covalent organic frameworks for synergistic photocatalytic hydrogen evolution. J. Mater. Chem. A **10**, 17691-17698 (2022). https://doi.org/10.1039/D2TA04748A
